# Meat-Borne Bacterial Pathogen Detection: Conventional, Molecular and Emerging AI-Based Strategies

**DOI:** 10.3390/diagnostics16091360

**Published:** 2026-04-30

**Authors:** Athar Hussain, Qindeel Abbas, Muhammad Nadeem, Aquib Nazar, Ali Athar, Hafiz Ubaid Ur Rahman

**Affiliations:** 1 School of Food and Agricultural Sciences (SFAS), University of Management and Technology (UMT), Lahore 54000, Pakistanm.nadeem@umt.edu.pk (M.N.); 2 Genomics and Informatics Lab (GIL), Ltd., Lahore 54000, Pakistan; 3 Department of Life Sciences, School of Sciences (SSC), University of Management and Technology (UMT), Lahore 54000, Pakistan; 4 Seoul St. Mary’s Hospital, Catholic University of Korea, 222 Banpo-daero, Seocho-gu, Seoul 06591, Republic of Korea

**Keywords:** artificial intelligence, meat-borne pathogens, antimicrobial resistance, genomic surveillance, phage therapy, biosensors, pathogen detection

## Abstract

Meat serves as a prime medium for the growth of foodborne pathogens due to its rich protein content and high water activity, contributing significantly to the global burden of foodborne illnesses. This review synthesizes current advances in meat-borne bacterial pathogen detection with particular emphasis on emerging artificial intelligence (AI)-enabled applications. Major pathogens of concern, including *Salmonella*, *Listeria monocytogenes*, *Escherichia coli*, *Campylobacter*, *Clostridium*, and *Staphylococcus aureus*, are examined in relation to their relevance across the meat supply chain. Recent progress in biosensors (clustered regularly interspaced short palindromic repeats), CRISPR-based assays, isothermal amplification, and metagenomics is evaluated alongside the growing role of AI in automating signal interpretation, enhancing image-based diagnostics, and supporting early contamination prediction. AI-based systems have proved 96.4–104% recovery and 100% bacterial capture ability. Embedding AI methods in a wet lab demands technical and logical modeling, as well as learning and calibration decorum. Nonetheless, AI readiness and full-scale application for meat-borne pathogens surveillance are on the way. Furthermore, additional focus is aligned on meat-borne bacterial pathogen genomic databases, i.e., (NCBI Pathogen Detection, EnteroBase, VFDB, ComBase, and GenBank), which serve as critical training resources for AI models for outbreak tracking, virulence profiling, and antimicrobial resistance (AMR) prediction. By integrating molecular methods, genomic surveillance, and AI-driven analytics, this review presents a framework for strengthening meat safety systems. This will improve early detection capabilities and support data-driven public health interventions in the future.

## 1. Introduction

Globalization and the surge in trade have rendered the food supply chain vulnerable to various challenges, including bioterrorism, food safety issues, and food fraud [1]. Most foodborne diseases result from poor food handling practices and limited or time-consuming detection techniques, which are either limited in sensitivity or too time-consuming, delaying timely intervention [2,3]. A substantial proportion of these illnesses originates from contaminated meat products [4]. Despite the longstanding debate surrounding vegetarianism and non-vegetarianism, evidence indicates that humans have consumed meat for over 2.6 million years, pre-dating the domestication of animals [5,6]. As of 2018, 1.8 billion people, representing 23.9% of the global population, consumed at least 100 g of meat daily [7]. Most meat consumed annually becomes infected with various pathogenic bacteria and viruses [8]. Viral contamination of foods, particularly meat, can cause massive outbreaks, with the contamination threshold often being fewer than 100 viral particles [9]. Since viruses require a live host and cannot grow in food on their own, the organoleptic properties of the food remain unchanged, posing a hidden threat of viral contamination [10]. However, this review focuses exclusively on meat-borne bacterial pathogens, which represent the primary contributors to global food-borne illnesses. A wide range of bacterial species, including *Salmonella*, *Listeria monocytogenes*, *Shigella*, *Escherichia coli*, *Campylobacter*, *Clostridium*, *Staphylococcus aureus*, *Pseudomonas*, *Vibrio* species, and *Yersinia*, infect meat and meat products worldwide [11,12]. Meat provides essential nutrients, although processed variants may contain preservatives that could pose health issues [13]. Despite conflicting evidence on the health effects of meat consumption, further research is needed to fully understand the health impacts, potential benefits, and metabolic effects of processed meats [14].

Over the past decades, pathogen detection in meat has progressed from traditional culture-based and microscopic methods to highly sensitive molecular, biosensor, and AI-enhanced platforms. Conventional techniques, including selective culturing and colony enumeration, remain foundational but are limited by long turnaround times and their inability to detect viable non-culturable cells [15,16,17,18,19]. Immunoassays such as ELISA improved speed and specificity, but face challenges related to antibody stability and cross-reactivity [20,21]. Molecular diagnostics, including PCR, multiplex PCR, LAMP, and NASBA, introduced rapid and precise nucleic-acid–based detection, enabling identification of pathogens like *Salmonella*, *Listeria*, and *E. coli* at low concentrations [22,23,24,25,26]. Recent advances in biosensors, including electrochemical, optical, piezoelectric, and nanotechnology-based devices, have further enhanced on-site and real-time detection capabilities with low CFU/mL (10^2^ CFU/mL) and short time [22,27,28,29,30,31,32]. Complementing these tools, modern CRISPR-Cas systems and metagenomics provide ultra-specific and culture-independent detection with high sensitivity, while whole-genome sequencing and surveillance databases (e.g., NCBI Pathogen Detection, EnteroBase, and VFDB) enable comprehensive tracking of virulence and antimicrobial resistance genes [33,34,35,36,37,38,39,40]. More recently, AI-driven models and imaging systems have emerged as powerful tools that automate colony classification, enhance biosensor signal interpretation, and predict contamination events, demonstrating accuracies above 95% for major meat-borne pathogens, enabling faster, data-driven food safety decisions [41,42,43,44,45,46,47]. However, many of these high-accuracy values are reported from studies conducted using relatively small or curated experimental datasets, thereby generating controlled laboratory conditions, which may not fully represent the biological and environmental variability encountered in industrial meat processing systems. Consequently, careful evaluation of dataset size, validation strategy (using varied datasets, biological matrices variability, robustness checks), and model generalization is essential to avoid overfitting and to ensure that AI-based detection systems remain reliable when applied to heterogeneous real-world meat samples.

Keeping in view these trends in pathogen detection, the current review focuses on meat-borne bacterial pathogens and the technological advancements used to detect and control them. It examines the major bacterial pathogens that are associated with meat, their physiological and pathological attributes, along with their role in contamination across the meat supply chain. Furthermore, it critically evaluates conventional, molecular, and advanced detection technologies, including biosensors, nucleic-acid assays, a CRISPR-Cas system, metagenomics, and emerging AI-enabled diagnostic frameworks that are enhancing sensitivity, speed, and predictive surveillance. Additionally, it also highlights the increasing importance of genomics and bioinformatics resources, such as NCBI pathogen detection, EnteroBase, ComBase, and Genebank, in supporting pathogen tracking, virulence profiling, and antimicrobial resistance monitoring and feeding data into advanced AI-based models. Collectively, the study integrates the latest developments to outline the evolving tools required to strengthen meat safety, improve outbreak detection, and support public health protection.

### Review Methodology

The current review was compiled using various search engines, i.e., NCBI, PubMed, ScienceDirect, Google Scholar, and alike. Only published research articles, reviews, case studies, and meta-analyses were selected, while unpublished, incomplete, and in languages other than English or irrelevant were excluded. The search strategy applied Boolean operators like “AND”, ”OR”, etc., along with search terms including meat pathogens, bacterial toxins, meat toxins, AI in pathogen surveillance, and meat infection, etc.

## 2. Epidemiology and Emerging Concern of Meat-Borne Bacterial Pathogens

### 2.1. Major and Emerging Meat-Borne Bacterial Pathogens

Meat-borne pathogens pose a significant threat to global public health, causing diseases primarily transmitted through the contamination of meat by bacteria [4,48]. The One Health approach is essential for implementing effective control strategies, as it recognizes the network of human, animal, and environmental systems in managing bacterial zoonotic pathogens associated with meat [49,50]. Historical outbreak analyses further highlight the burden of meat-borne diseases, like in 1980 and 2015, *E. coli* and *Salmonella* were identified as the leading causes of meat-associated outbreaks, accounting for 33 and 21 events, in Europe and the United States, respectively [12]. In these outbreaks, the fresh and processed meat products were observed as the cause of *E. coli* outbreaks, whereas raw, cured, and fermented sausages were found with *Salmonella* outbreaks [12]. Later, another study critically explored the connection between animal farming, meat consumption, and the emergence of infectious diseases in humans, highlighting that meat production increases epidemic risks through more significant contact with affected animals and environments [51]. In addition to these, several other meat-borne pathogens, including *Clostridium*, *Staphylococcus*, and *Campylobacter*, also contribute to significant foodborne disease through potent toxins such as botulinum neurotoxins, staphylococcal enterotoxins, and perfringolysin (a toxin that is prevalent in infected or unhealthy meat after slaughtering and acts by binding cholesterol in the host cell membrane, creating pores, and ultimately cell death) [52].

Beyond these well-established pathogens, several newly emerging bacteria are increasingly implicated in meat contamination and foodborne illness [53]. While traditional pathogens, such as *Salmonella* and *E. coli*, remain major contributors to disease burden. Further, pathogens, including *Arcobacter* spp., *Cronobacter sakazakii*, *K. pneumoniae*, and *B. cereus*,have gained rising epidemiological significance in recent years [54,55]. *Arcobacter* spp. have been detected in more than 20% of carcasses and meat products, being recognized as cause of entire and prolonged gastrointestinal illness [56]. These have also demonstrated higher tolerance to common preservation processes than *Campylobacter*, enabling sustained contamination within the meat supply chain [52]. Although *C. sakazakii* has historically been linked to powdered infant formulae, increasing reports from Europe and Asia have implicated that ready-to-eat meat products can cause severe infections, such as meningitis, septicemia, or necrotizing enterocolitis, particularly among immunocompromised individuals [15,57,58,59]. Furthermore, *K. pneumoniae* has also emerged as a food-borne pathogen due to its presence in raw meat. This is inherent in water, soil, and farm vicinity, as well as in the GI tract of livestock, where it can pose a health threat due to poor handling of meat animals [60]. In another study, *K. pneumoniae* was detected in 12% of meat samples, and its persistence within the processing environment represented its biofilm-forming capacity, making the elimination process less effective [61,62,63]. Likewise, *B. cereus*, though well-described in non-meat dishes, has now been increasingly reported in different meat products. It is reported in the literature that a few strains of *B. cereus* produce heat-stable toxins which remain active even at higher temperatures, like cooking of meat, causing severe gastrointestinal disease [64,65]. Their growing detection in raw and ready-to-eat meat, together with their persistence in diverse environments, signals a shift in meat-borne pathogen epidemiology and underscores the need for stronger surveillance and improved detection technologies. Building on this need, future studies are likely to explore these toxins as identification markers for advanced tools such as biosensors and AI-supported diagnostic systems.

### 2.2. Prevalence of Meat-Borne Pathogen-Associated Diseases

The global prevalence of meat-associated bacterial infections remains substantial, and is driven by contamination of animal products during slaughter, processing, and distribution. Surveillance reports demonstrated that *Salmonella*, *E. coli*, *L. monocytogenes*, *Campylobacter*, and *Y. enterocolitica* are consistently detected across diverse meat commodities, reflecting their ability to persist in production environments and enter the food chain through multiple contamination routes [66,67]. Of these, *Salmonella* remains one of the most frequently detected contaminants in meat products, colonizing the gastrointestinal tracts of livestock and spreading widely during slaughtering and processing. Its ability to form biofilm, particularly in poultry facilities, enables persistence on equipment and surfaces, thereby increasing the likelihood of carcass contamination [68,69]. The spread of *E. coli*-associated diseases has been linked to beef, poultry, fish, and contaminated water sources, with diarrheal disease resulting from ingestion of even low infectious doses [70,71]. Nonetheless, *L. monocytogenes* remains a particular pathogen of global concern, contaminating meat, poultry, seafood, dairy products, vegetables, and fish. The incidence of Listeriosis, which is caused by *L. monocytogenes*, has been rising in the food industry because of its capacity to survive during refrigeration and sanitation procedures, contributing to contamination of meat and ready-to-eat products [72]. *Campylobacter* contamination is often introduced during poultry defeathering and evisceration, whereas *Y. enterocolitica* spreads primarily through pork processing, especially when cross-contamination occurs during carcass handling. These epidemiological patterns highlight pathogen-specific contamination points within the meat chain, underscoring the need for robust monitoring systems capable of detecting early, low-level contamination events.

**Figure 1:**
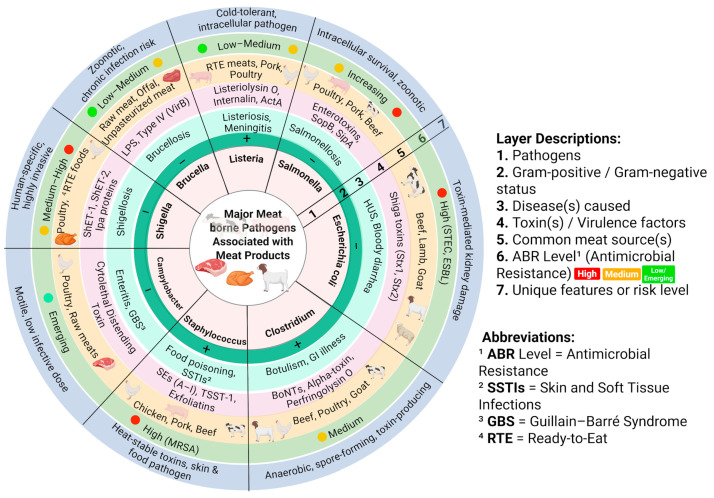
Pathogen Diversity, Toxins, and Virulence Factors. Provides a circular schematic overview illustrating the diversity and epidemiological relevance of major meat-borne bacterial pathogens. The figure highlights key attributes, including Gram-staining profiles, major toxins (e.g., *Stx1*/*Stx2* in *E. coli*, ListeriolysinO in *Listeria monocytogenes*, enterotoxins in *B. cereus*, and virulence secretion systems in *Salmonella*), common meat sources (poultry, beef, pork, fish), AMR levels, and unique ecological or epidemiological features.

Certain population groups, including infants, young children, older adults, pregnant women, and immunocompromised individuals, experience disproportionately severe outcomes from meat-borne bacterial infections [73,74]. Their heightened susceptibility, combined with the low infectious dose of several pathogens such as *L. monocytogenes* and Shiga toxin–producing *E. coli*, underscores the need for early and highly sensitive detection systems in meat products [75]. Rapid identification of contamination before distribution is therefore essential to prevent severe disease in these high-risk groups (Figure 1).

In terms of public health, the detection of meat-borne pathogens is important not only for identifying microorganisms but also for preventing human diseases and controlling outbreaks. Fast and sensitive detection technologies are important for breaking the chain of transmission in the farm-to-folk continuum by identifying products that are contaminated early, before they reach consumers. Late or inadequate diagnosis may result in mass outbreaks, high hospitalization levels, and serious challenges, especially in high-risk groups like immunocompromised persons, pregnant women, and the elderly. Thus, any positive change in detection, sensitivity, and turnaround time is directly proportional to the increased use of food safety surveillance systems.

## 3. AMR in Meat-Borne Pathogens and Their AI-Based Risk Assessment

### 3.1. Prevalence and Impact of Antibiotic Resistance in Meat-Borne Pathogens

Antimicrobial resistance (AMR) in meat-borne pathogens has become a major global health concern because it directly affects food safety and disease management. The extensive use of antibiotics in livestock production accounts for almost 67% of global antibiotic consumption. This level of use creates strong selective pressure, accelerating the development of multidrug-resistant (MDR) bacteria in animal production systems. Key pathogens such as *Salmonella*, *Escherichia coli*, *Campylobacter*, and methicillin-resistant *Staphylococcus aureus* (MRSA) are now frequently detected in meat products and often show resistance to several classes of antimicrobials, causing consumers’ health risks [76,77]. The prevalence of AMR varies across regions, depending on regional factors and bacterial species identified. For example, antibiotic-resistant *Salmonella* is responsible for nearly 13% of reported cases in the United States and is linked to more severe illness and higher hospitalization rates. In Southeast Asia, isolates of *S. Typhimurium* commonly show resistance to ampicillin, tetracycline, and fluoroquinolones [78]. Shiga-toxigenic *E. coli* strains are resistant to β-lactams and fluoroquinolones, where this situation further complicates treatment, while MRSA contamination, reported in about 5% of raw meat samples in Europe, raises increasing concern about zoonotic transmission [79,80]. Some methicillin-resistant *Staphylococcus aureus* (MRSA) have started causing zoonotic problems, with some evidence proving transmission from livestock to humans *via* meat products [81]. Recent information indicates that MRSA could be traced to nearly 5% of raw meat samples in Europe, thus calling for the urgent use of alternative antimicrobial strategies [82].

Artificial intelligence (AI) is now an important tool for improving the early detection and prediction of AMR within meat production systems [83]. AI models can combine diverse data sources such as antibiotic usage records, slaughterhouse microbiological findings, whole-genome sequencing data, and environmental information [83,84,85]. This integration allows AI systems to identify resistance genes, efflux pump activity, and drug-modifying enzyme patterns, which helps to predict the likelihood of AMR contamination before meat reaches consumers [85,86].

### 3.2. Alternative Strategies to Combat Antibiotic Resistance

The rising burden of AMR in meat production, highlighted by AI-based surveillance systems, has increased interest in alternative antimicrobial strategies that reduce dependence on conventional antibiotics [87]. Phage therapy is one promising option. Bacteriophages can selectively infect and kill bacterial pathogens without encouraging resistance [88]. They can be added to animal feed, used in processing areas, or applied directly to meat products [89]. Studies have shown that phage treatment can significantly reduce *Salmonella* contamination in poultry and beef [90]. Antimicrobial peptides (AMPs) also represent an important alternative. These naturally occurring or synthetic molecules have broad antibacterial activity and can function as food preservatives, feed additives, or therapeutic agents in veterinary medicine [91]. Synthetic AMPs have achieved reductions of more than 90% in *E. coli* populations in meat samples [92,93]. Plant-derived antimicrobial compounds, probiotic interventions, and targeted vaccination strategies offer additional ways to limit pathogen growth and reduce antibiotic use in livestock systems [94,95].

**Figure 2:**
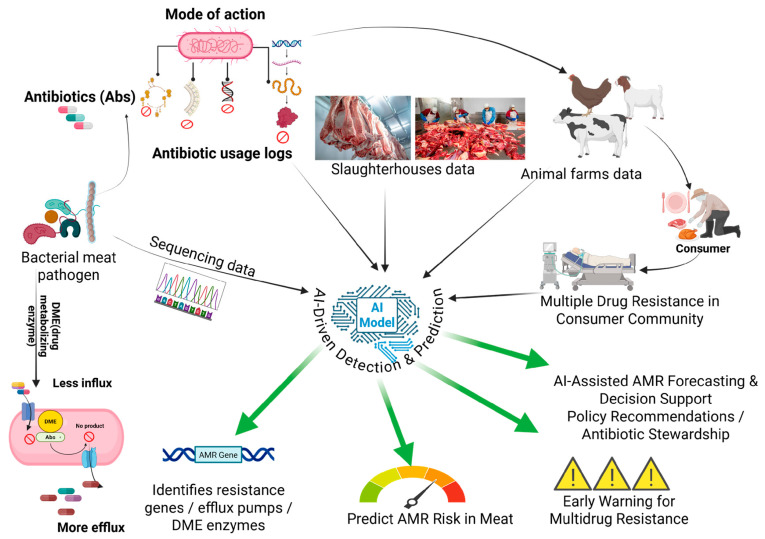
An AI-integrated framework illustrates how antibiotic use, bacterial pathogens, and resistance mechanisms in the food animal production chain contribute to antimicrobial resistance (AMR) in consumers. The model incorporates on-farm data, slaughterhouse information, antibiotic usage logs, and pathogen sequencing outputs to enable AI-driven AMR detection, thereby predicting resistance genes and efflux/DME mechanisms. This also aids in the estimation of AMR risk in meat and in public health forecasting for early-warning and stewardship interventions.

AI contributes further by improving the design, optimization, and selection of these alternative strategies. AI-assisted phage matching helps to identify phages that work effectively against new resistant strains [96]. Machine-learning models support AMP research by predicting antimicrobial activity, stability, and safety for thousands of potential peptide sequences [97]. AI-guided chemical screening assists in discovering plant-derived compounds with strong antimicrobial properties, while computational models simulate how probiotic strains interact within the gut or meat environment to identify those most likely to inhibit pathogens [98,99]. AI-based vaccine modeling also enhances the evaluation of immune responses in livestock and helps to design more efficient vaccination programs. Through these advances, AI not only improves AMR surveillance but also accelerates the development of innovation in microbial control strategies, contributing to a more sustainable and resilient approach to AMR management in food systems (Figure 2).

## 4. Meat-Borne Bacterial Pathogen Detection: Conventional to Advanced Molecular Approaches

### 4.1. Conventional Methods

Microbial culture remains a fundamental approach for the detection of meat-borne bacterial pathogens, based on the growth of microorganisms on selective and differential media to allow their isolation and identification. Standard plating techniques include pour, spread, and streaking, typically requiring 24–72 h of incubation, depending on the target organisms in labs [100,101]. After incubation, bacterial colonies are examined for morphological characteristics and are subsequently confirmed using biochemical assays for the detection of key foodborne pathogens, including *Salmonella* spp., *L. monocytogenes*, and *E. coli* [102,103]. Reported detection limits for culture-based assays in meat matrices generally range from 10^3^ to 10^4^ CFU/mL, with diagnostic accuracy being reported between 80 and 90% under controlled laboratory conditions [104]. Despite their reliability and regulatory acceptance, culture-based detections are limited by prolonged incubation periods, intensive labor requirements, and the inability to recover viable but non-culturable (VBNC) cells. To address these constraints, recent developments have focused on integrating automated imaging and computational analysis into culture-based workflows. Digital colony imaging systems and growth-monitoring software enable more consistent colony enumeration, minimize observer-dependent variability, and support earlier assessment of microbial growth trends [105,106]. Such refinements improve analytical efficiency and reproducibility while maintaining the established role of culture-based methods in routine pathogen surveillance.

Microscopy-based techniques provide direct visualization and quantitative assessment of bacterial contamination on meat and meat-contact surfaces [107,108]. Conventional fluorescent staining methods, such as DAPI-based direct cell counts, are widely used to estimate total bacterial load, typically achieving detection limits of approximately 10^3^ CFU/mL [109,110]. These approaches have been expanded through flow cytometry, viability staining, and scanning electron microscopy (SEM) to enable rapid enumeration, assessment of cell integrity, and visualization of bacterial attachment and biofilm formation. SEM and fluorescence-based methods offer high spatial resolution and detailed structural information, particularly for studying surface-associated contamination; however, reported accuracy values generally range between 80 and 88%, depending on staining efficiency and sample preparation quality [111]. Their routine application is limited by high instrumentation costs, extensive sample preparation, and operator-dependent interpretation, particularly when distinguishing viable cells from non-viable cells. Recent methodological refinements incorporate automated image quantification and standardized analysis pipelines to improve contrast resolution, reduce background noise, and enhance consistency across microscopic datasets [112,113]. These approaches improve cell-discrimination reliability and analytical throughput, supporting more reproducible assessment of microbial contamination patterns. As a result, microscopy-based detection continues to serve as a valuable complementary tool within conventional pathogen-detection frameworks for meat safety research and monitoring.

### 4.2. Advanced Non-Molecular Approaches for Meat-Borne Pathogen Detection

#### 4.2.1. Immunoassay-Based Methods

Immunoassays continue to play a major role in food pathogen detection, with recent innovation emphasizing rapid analysis, multiplexing capability, and nanomaterial enhancement rather than reliance on the conventional enzyme-linked immunosorbent assays (ELISA) alone [114,115,116]. Nanomaterial-based ELISAs (nano-ELISA) have significantly improved analytical sensitivity and signal stability, making them more suitable for complex food matrices such as meat products [20]. Several studies demonstrated the use of monoclonal antibody-based assays for rapid detection of *Salmonella* across diverse food systems, while fluorescence immunoassays provide high sensitivity for detecting antigens, drugs, and hormones [21]. For instance, a label-free immunofluorescence strip sensor incorporating FITC enables rapid detection of *E. coli O157:H7*, presents visual detection limits comparable to conventional ELISA, reducing assay time [117].

Modern food-diagnostic immunoassays now employ magnetic beads, quantum dots, gold nanoparticles, and lateral-flow fluorescence strips to achieve single-or-sub-picogram sensitivity for major meat-borne pathogens, including *E.coli O157:H7*, *Salmonella*, *Staphylococcus*, and *Listeria* spp. [20,118,119]. These nano-ELISA platforms offer substantially lower detection limits than conventional toxin-based assays while supporting multiplex analysis within a single reaction format [117]. Despite these advances, immunoassays remain susceptible to cross-reactivity and antibody instability, particularly when applied to heterogeneous meat matrices. Hence, careful antibody selection, validation against non-target organisms, and matrix-specific optimizations are essential to minimize false-positive results to ensure reliability in routine meat safety testing [116,120] (Table 1).

#### 4.2.2. Biosensor-Based Detection

Biosensors have emerged as a powerful class of non-molecular detection platforms for meat-borne bacterial pathogens, offering rapid response time, high sensitivity, and cost-effective operation compared to traditional microbiological methods [121]. Intelligent sensor platforms enable low-cost, portable, rapid, and real-time analysis, strengthening global food safety monitoring through direct detection of biological or biochemical signals [122]. Since the introduction of the first enzyme electrode by Clark, Biosensor technology has evolved substantially, leading to the development of diverse sensing platforms optimized for food and meat safety applications [123]. Biosensors used for meat-borne pathogen detection are typically classified by their bioreceptors, including enzymes, antibodies (immunosensors), nucleic acids (genosensors), whole cells, and aptamers [124]. Each recognition element presents distinct advantages and limitations with respect to selectivity, stability, and compatibility with complex meat matrices [125]. While natural receptors provide high specificity towards target bacterial pathogens, their susceptibility to degradation has driven the development of artificial recognition elements, such as molecularly imprinted polymers (MIPs), or “plastic antibodies,” which offer improved stability and extended operational lifetimes under harsh meat-processing conditions [126].

Biosensor platforms combine biological components with electronic components via a transducer that converts bacterial binding events into quantifiable signals [127,128]. Optical biosensors have demonstrated strong performance in the meat-processing and food control due to their high sensitivity, robustness, and minimal sample preparation requirements. For example, an optical biosensor utilizing antibody-functionalized porous silicon film enabled the rapid identification and quantification of *E. coli* in complex food industry processes, demonstrating real-time detection and high specificity [129]. Piezoelectric biosensors exploit mass-induced frequency changes at sensor surfaces and have been successfully applied for detecting bacterial toxins and contaminants relevant to meat safety. A label-free quartz crystal microbalance immunosensor demonstrated sensitive detection of Aflatoxin B_1_ in food samples across a wide linear range [130,131]. Electrochemical biosensors are particularly attractive for meat-borne pathogen detection due to their portability and rapid signal generation [132]. A gold-nanoparticle-modified carbon screen-printed electrode enabled label-free detection of *E. coli O157:H7*, supporting rapid, field-deployable diagnostics in the meat testing workflow [27,133]. Furthermore, magnetic nano-beads have been extensively utilized in biosensor development, particularly for magnetic separation, target labeling, and signal amplification, with applications in biomedical diagnostics, food safety, and environmental monitoring [134]. Notably, a LAMP-CRISPR/Cas12a-based biosensor using TEPA-functionalized magnetic nanoparticles achieved a detection limit of 100 CFU/per mL for *L. monocytogenes*, highlighting how biosensors can be effectively adapted for ultra-sensitive detection of meat-borne bacterial pathogens when coupled with amplification technologies [135]. In conclusion, biosensors play a crucial role in meat-borne bacterial pathogen detection by providing rapid, sensitive, and potentially on-site diagnostic capability. These constraints explain why biosensor-based systems currently complement rather than replace culture-based reference methods in routine meat safety surveillance, underscoring the need for continued optimization and standardization [136,137].

#### 4.2.3. Nanotechnology in Pathogen Detection

Detection methods that use nanotechnology enhance the sensitivity and specificity of biosensors [31]. Gold nanoparticles (AuNPs) and quantum dots (QDs) are widely applied in lateral flow assays and electrochemical sensors for quick detection of pathogens such as *L. monocytogenes* and *C. jejuni* [138,139]. A 2020 study demonstrated that AuNP biosensors detected *Salmonella typhimurium* from pork meat in a short time in Korea, as opposed to the traditional culture methods, which need 24–48 h for confirmation [29,140]. Nanotechnology-based biosensors containing gold nanoparticles and quantum dots make it possible for the real-time detection of pathogens with attractive specificity. Functionalization to improve lateral flow assays and electrochemical sensors has been used extensively to enhance the detection sensitivity to *Salmonella* and *E. coli*. Metagenomics helps to identify both culturable and non-culturable pathogens by sequencing all genetic material from a sample, thus assisting with tracing contamination sources and understanding microbial ecology [141]. Portable devices for biosensing that use surface plasmon resonance (SPR) and electrochemical impedance spectroscopy (EIS) would enable rapid detection on-site, thereby reducing reliance on laboratory-based testing. In 2021, AI models were employed to predict the outbreak of *S. enterica* in processed meat in Canada, utilizing historical patterns of outbreaks and genomic sequencing data to initiate early recalls, thereby reducing public health impact [139].

#### 4.2.4. Surface-Enhanced Raman Spectroscopy

**Figure 3:**
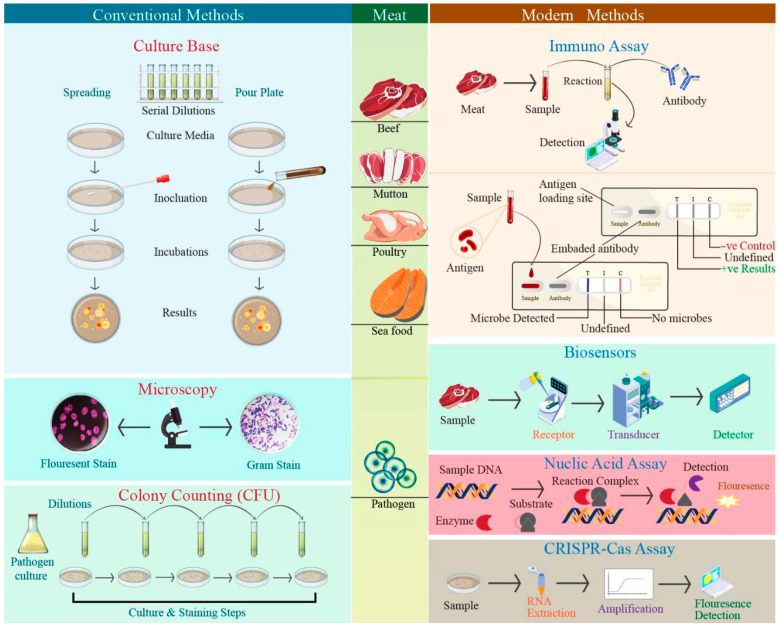
Comparison of conventional and modern methods for meat pathogen detection. The left side depicts conventional techniques, including culture-based methods (spreading, serial dilutions, and inoculation), microscopy (fluorescent and Gram stains), and colony counting (CFU). The right side presents modern methods, such as immunoassays using antibodies for antigen detection, biosensors, nucleic acid assays for DNA-based detection, and CRISPR-Cas assays for RNA extraction, amplification, and fluorescence detection. Various meat types (beef, mutton, poultry, and seafood) are used as samples across these detection techniques.

The ever-changing and exciting non-destructive methods, such as Raman spectroscopy and hyperspectral imaging, provide rapid identification of pathogens without ever destroying the sample. Surface-enhanced Raman spectroscopy (SERS) has shown the detection of *S. aureus* biofilms with a sensitivity of 10^3^ CFU/mL [142,143]. On the other hand, hyperspectral imaging differentiates fluorescence in some meat products and changes with spoilage, using near-infrared (NIR) analysis to present an automatic quality assessment. It will be crucial to explore alternative approaches to combat AMR through phage therapy and AMPs, integrated with predictive AI risk modeling and the One-Health concept, to ensure global food safety [143]. Standardization and regulatory alignment are two key matters that ought to be addressed so as to optimize detection methodologies and response strategies. As illustrated in Figure 3, modern pathogen detection approaches integrate molecular diagnostics, biosensor platforms, and AI-driven analytics, enabling faster and more sensitive detection compared with conventional culture-based techniques (Figure 3).

### 4.3. Advanced Molecular Approaches for Meat Pathogen Detection

#### 4.3.1. Nucleic-Acid-Based Methods

In recent years, the food industry has increasingly incorporated nucleic acid-based approaches for rapidly and sensitively identifying pathogenic bacteria, significantly improving food safety and quality assurance [144]. Nucleic acid-based techniques have undergone the most rapid modernization. Methods like polymerase chain reaction (PCR), recent applications in meat safety emphasize digital PCR (dPCR), multiplex PCR, and rapid isothermal amplification techniques, which have enabled absolute quantification of low-abundance pathogens and AMR-genes in meat samples [144]. Multiplex PCR (mPCR) is useful for detecting various pathogens or assessing different virulence factors within a single pathogen, such as *Escherichia coli*, where mPCR can differentiate pathogens based on their pathotype or serotype using distinct amplicon profiles [145]. However, mPCR can be labor- and cost-intensive, making it less suitable for field operations. In contrast, loop-mediated isothermal amplification (LAMP) has gained popularity due to its shorter reaction times, sensitivity, specificity, and independence from specific equipment. LAMP is also less susceptible to sample inhibitors, making it a practical tool for detecting pathogens in raw food samples without extensive preparation [146]. Additionally, nucleic acid sequence-based amplification (NASBA) is an isothermal RNA amplification method, particularly effective for detecting microbial pathogens in food and environmental samples [147]. By targeting 16S rRNA and mRNAs, NASBA allows for the specific identification of viable cells [26,148]. These nucleic acid-based methods, including PCR, mPCR, LAMP, and NASBA, represent key innovations that enhance the speed, sensitivity, and accuracy of pathogen detection in food safety testing [149]. While the molecular diagnostic techniques are very helpful in quantifying the pathogen load in the sample, their efficiency is hindered owing to the requirement of well-trained handlers, sample preparation, time consumption, cost effectiveness, and presence of inhibitors in food samples [150].

#### 4.3.2. CRISPR-Cas-Based Methods

CRISPR-enabled molecular diagnostics has rapidly advanced in high-sensitivity and field-deployable tools that are capable of detecting bacterial pathogens in meat and food processing environments with short turnaround times [151]. The CRISPR-Cas-based method has emerged as a promising tool for pathogen nucleic acid detection, overcoming the limitations of traditional detection methods by offering enhanced specificity and sensitivity. One example of its application is developing a rapid nucleic acid detection-based method for *E. coli* O157:H7 using the CRISPR/Cas12a system targeting the *rfbE* gene. This system achieved an impressive sensitivity of 0.9 pg/μL for DNA and 6.5 × 10^4^ CFU/mL for bacterial concentrations [152]. Another significant advancement is the CRISPR/Cas12a-based fluorescence detection platform, “Cas12aFDet,” developed for the rapid and specific detection of *L. monocytogenes* serotype 4c. This platform integrated PCR or recombinase-aided amplification (RAA) methods with Cas12a-mediated cleavage, achieving detection within 15 min and avoiding amplicon contamination. It demonstrated a sensitivity of 3.37 × 10^1^ CFU/mL for PCR-based detection and 1.35 × 10^2^ CFU/mL for RAA-based detection. These advancements show the potential of CRISPR-Cas systems in revolutionizing pathogen detection with rapid, sensitive, and specific results. These techniques provide significant accuracy in quantifying and detecting pathogens; however, factors like off-target effects during genome editing and the complexity of CRISPR-Cas classification can hinder standardization and need integration with amplification methods like PCR, inducing additional steps as limiting factors [38] (Figure 2).

These detection technologies have clinical significance because they can be used to give timely and accurate detection of pathogens, which is very crucial in preventing the entry of contaminated meat into the food supply chain. Technologies that can prove a reduction in detection time from days to hours contribute significantly to the reduction in outbreaks. In addition, extremely sensitive procedures that can identify low levels of contamination are especially valuable in the prevention of infections by pathogens that cause infections at low dosages, i.e., *L. moncytogeneses* and *E. coli*. As a result, the development of such detection technologies not only improves laboratory diagnostics but also enhances the level of safety of the population and the control of food safety.

#### 4.3.3. Metagenomics for Culture-Independent Identification of Pathogens

Metagenomics allows the detection of a myriad of pathogens through the sequencing of all DNA or RNA present in the sample [153]. The method is advantageous for identifying pathogens that are non-culturable or previously unknown. In 2019, a metagenomic study of poultry meat conducted in Germany found *Campylobacter* contamination levels exceeding 30%, leading to stricter hygiene standards being imposed by regulatory bodies [154]. Thus, on-site pathogen detection became a reality with portable biosensors using surface plasmon resonance (SPR) and electrochemical impedance spectroscopy (EIS) [155]. These devices have detection limits as low as 10^2^ CFU/mL for *E. coli* O157:H7 in meat matrices [156] (Table 1).

Although there is a broad spectrum of detection technologies that have been developed against meat-borne pathogens, the technologies perform quite differently in terms of sensitivity, their complexity of operation, the skilled staff, complex meat matrices, and their applicability in the real world. Traditional culture-based technologies are gold standards with good reliability. These can verify viable organisms requiring a long turnaround time (24–27 h), which restricts their usefulness in making quick decisions in an industrial setting. On the other hand, molecular methods, including PCR and isothermal amplification, have much higher sensitivity and shorter time to detect pathogens, even at low concentrations (10^2^ CFU/mL). Conclusively, the latter are beneficial in terms of sensitivity, accuracy, and the shortest reporting time with a futuristic scope in meat safety.

**Table 1:** Comparative overview of pathogen detection technologies in the meat industry: evolution from conventional to AI-enhanced approaches.

Category/Detection Approach	Principle/Example	Target Meat Pathogens Detected	Detection Time	Sensitivity/Detection Limit	Accuracy/Specificity	Cost/Test ($) r	Field/On-site Applicability	Major Limitations	AI-Added Benefit/Enhancement	References
I. Conventional Approaches										
Culture-based Methods	Growth on selective media (pour, spread, streak plate)	*Salmonella* spp., *E. coli* O157:H7, *Listeria monocytogenes*, *Campylobacter* spp.	24–72 h	10^3^–10^4^ CFU/mL	80–90%	$2–10	No (lab only)	Fails to detect VBNC microbes; media bias	Faster automated counting	[100,101,102,103,105,106]
Microscopy-based Detection	SEM/Fluorescent staining (DAPI, epifluorescence)	*E. coli*, *Listeria* spp., *Staphylococcus aureus*	6–24 h	~10^3^ CFU/mL	80–88%	$20–100	No	Laborious sample prep; dependent on stain binding	Improved image clarity	[109,110,111]
II. Advanced Non-Molecular Approaches										
Immunoassay (ELISA, Nano-ELISA)	Antibody–antigen binding with colorimetric/fluorescent readout	Salmonella, *E. coli*, Listeria, Campylobacter	3–6 h	10^2^–10^3^ CFU/mL	90–95%	$10–25	Lab or field (strips)	Cross-reactivity, antibody instability	Reduced false positives	[20,114,115,116,117,118,120,129,157]
Biosensor-based Detection	An enzyme, antibody, DNA, or aptamer-based sensor with a transducer	Salmonella, Listeria, *E. coli*	30 min–2 h	10^2^ CFU/mL	95–98%	$5–30	Yes (portable)	Bioreceptor instability; background noise	Enhanced signal filtering	[29,121,124,128,132,158]
Electrochemical Biosensor	AuNP-modified electrode for *E. coli* O157:H7	*E. coli* O157:H7, *Salmonella* spp., *Listeria monocytogenes*	~1 h	10^2^ CFU/mL	96–98%	$3–15	Yes	Surface fouling; short lifespan	Improved signal stability	[27,132,159]
Optical/Piezoelectric Biosensor	Light or frequency change via antigen–antibody interaction	*E. coli*, Salmonella, Listeria, *S. aureus*	<1 h	10^2^ CFU/mL	96–98%	$10–40	Yes	Signal drift, calibration required	Better drift correction	[129,160,161]
Nanotechnology-based Sensors	AuNPs, QDs, SPR, EIS biosensors for rapid pathogen detection	*E. coli*, Salmonella, Listeria, *S. aureus*	10–15 min	10^2^ CFU/mL	97–99%	$2–20	Yes (on-site)	Nanoparticle stability, aggregation	Improved pattern recognition	[30,31,32]
III. Molecular Approaches										
PCR/qPCR	DNA amplification using a thermocycler	*E. coli* O157:H7, *Salmonella* enterica, *Listeria* monocytogenes, *Campylobacter jejuni*	4–6 h	10^2^–10^3^ CFU/mL	95–98%	$15–50	Limited field use	Needs a skilled operator; inhibitors in samples	Faster curve analysis	[17,23,24,25,161,162]
Multiplex PCR (mPCR)	Simultaneous amplification of multiple targets	Multiple pathogens simultaneously	3–5 h	10^2^ CFU/mL	96–98%	$20–60	No	Primer optimization complex	Optimized primer design	[22,23,24]
LAMP/NASBA	Isothermal DNA/RNA amplification	*Salmonella*, *E. coli*, *Listeria*	1–2 h	10^2^ CFU/mL	96–99%	$5–20	Yes (field-suitable)	Primer design critical	Cleaner signal reading	[26,135,146]
CRISPR-Cas-Based	Cas12a-mediated cleavage post-amplification	*E. coli*, *Salmonella*, *Listeria*	15–30 min	3.4 × 10^1^–10^2^ CFU/mL	98–99%	$10–40	Portable (POC)	Off-target cleavage; multi-step	Improved fluorescence readout	[33,34,35,37,38,151,152]
Metagenomics (WGS/Shotgun)	Sequencing of all DNA/RNA for pathogen ID	All detectable pathogens	6–12 h	Detects non-culturable	>98%	$100–500	No	Expensive; bioinformatics required	Faster sequence sorting	[141,153,154,163]

## 5. Significance of Genomic Databases in Meat-Borne Bacterial Pathogen Research and Surveillance

### 5.1. NCBI Pathogen Detection System

The NCBI Pathogen Detection system is one of the most important genomics surveillance platforms for foodborne bacteria, integrating whole genome (WGS) data from clinical, environmental, and food sources to support real-time pathogen tracking and antimicrobial monitoring [164]. Its relevance to meat safety is substantial, as the database initially prioritized four major meat-associated pathogens, including *Salmonella*, *E. coli*, *Shigella*, and *Listeria*, which remain key contributors to food-borne outbreaks globally [165]. The system has since expanded to include more than 50 taxa of public health significance, enabling broad genomics comparisons and cluster detection across diverse food-production environments. For meat-borne pathogens, NCBI Pathogen Detection provides a high-resolution framework for identifying outbreak-related strains, characterizing AMR profiles, and detecting emerging lineages linked to livestock and meat products. For example, a large-scale WGS analysis revealed that approximately 60% of sequenced isolates in the database originated from meat or meat-processing environments, with more than 147,000 resistance-associated genes detected across *Salmonella*, *Campylobacter*, *E. coli*, and *Listeria* strains [164]. Another investigation of the meat-associated *Salmonella* serovar I 4,[5],12:i:- analyzed 13,612 genomes and demonstrated that 71% of global isolates harbored the metal-tolerance island SGI-4, while 55% carried the *ASSuT* resistance pattern; notably, swine-derived strains exhibited the highest prevalence of multidrug resistance [166]. These findings highlight the database’s value in tracking the dissemination of virulent and resistant clones through the meat-production system.

Beyond surveillance, the NCBI Pathogen Detection system also serves as a critical source of high-quality WGS datasets that are increasingly used to train machine-learning models for meat-borne bacterial pathogen detection, source attribution, and AMR prediction. However, its broader utility is constrained by well-documented metadata and harmonization challenges. Inconsistent or incomplete sample annotation, such as missing production-stage information or environmental context, limits downstream comparative analyses and reduces model accuracy [167]. Unequal global sequencing capacity and limited cross-border data create surveillance gaps, especially for meat-associated lineages circulating in low-resource settings [168]. Additionally, differences in sequencing workflows and the lack of full interoperability with platforms such as GenomeTrakr, EnteroBase, and PulseNet further hinder integration of genomic, epidemiological, and food-chain metadata into unified detection pipelines [168]. These limitations collectively weaken the performance of AI-driven detection frameworks and restrict global outbreak-tracking capabilities. Strengthening metadata standards, unifying pathogen identifiers, and enhancing international data-sharing agreements are therefore essential to fully leverage NCBI Pathogen Detection for next-generation as well as AI-supported meat-borne pathogen surveillance systems (Table 2).

### 5.2. Virulence Factor Database (VFDB) for Gene-Level Profiling of Meat-Associated Pathogens

The Virulence Factor Database (VFDB), established in 2004, is a comprehensive resource dedicated to bacterial pathogens’ virulence factors (VFs). It provides detailed insights into the major virulence factors, including their structural features, functions, and mechanisms by which pathogens induce diseases [40]. Its relevance to meat-borne pathogens is well established, as VFDB enables systematic profiling of virulence determinants that shape pathogenicity, transmission potential, and outbreak severity. For instance, comparative WGS analysis of 243 *Salmonella* isolates from human and animal sources in China identified 670 virulence-associated genes, with VFDB enabling their classification into 14 functional categories most related to adherence, secretion systems, immunomodulation, and toxin production, which together accounted for over 84.63% of the detected virulence repertoire [169]. The VFDB further takes part in a leading role in understanding the prevalence of *Clostridium perfringens IRMC2505A*, a gastrointestinal pathogen linked to contaminated meat. VFDB-supported annotation detected major toxins, including phospholipase C, perfringolysin O, collagenase, and hyaluronidase, alongside multiple AMR determinants when cross-referenced with CARD [170]. Such gene-level resolution is essential for identifying high-risk clones and assessing the pathogenic potential of strains circulating within meat-production systems [171]. The VFDB is a foundational resource explicitly used in the literature for training machine-learning models, such as Virulent Hunter and various Deep Transfer Learning (DTVF) frameworks, which aim to predict and classify bacterial virulence factors (VFs) and illuminate pathogenicity in diverse microbial contexts. For meat-borne pathogen detection, these VFDB-trained models are critical for differentiating highly virulent strains such as specific *Salmonella* serotypes or Shiga toxin-producing *E. coli* from benign environmental isolates [172]. However, the models’ utility in real-time food safety surveillance is fundamentally constrained by the VFDB’s own issues with heterogeneity in gene nomenclature and variable curation depth across taxa. These inconsistencies reduce the models’ generalizability and impede the rapid, accurate risk assessment necessary to identify the most dangerous contaminants in the meat supply chain.

### 5.3. EnteroBase Genomic Repository for High-Resolution Typing of Meat-Associated Pathogens

EnteroBase is a comprehensive genome database and web-based platform dedicated to foodborne pathogens, focusing on genera such as *Salmonella*, *Escherichia*/*Shigella*, *Clostridioides*, *Vibrio*, and *Yersinia* [173]. It integrates strain-level metadata, assembled genomes, cgMLST/MLST (core genomic multilocus sequence typing) profiles, and HierCC clustering outputs derived primarily from Illumina short-read sequencing, enabling consistent and recombination-robust genotyping across global datasets [174,175]. As of the latest update, EnteroBase hosts a vast collection of 1,026,432 bacterial strains, including 452,734 *Salmonella* strains, 293,315 *Escherichia*/*Shigella* strains, 30,388 *Clostridioides* strains, 17,118 *Vibrio* strains, and 8436 *Yersinia* strains, representing one of the world’s most comprehensive repositories for meat-associated pathogens [173]. For meat safety surveillance, EnteroBase has proven critical in tracing the diversity and epidemiology of *S. enterica* recovered from livestock and meat-processing environments. A Brazilian study analyzing bovine carcass isolates used EnteroBase MLST data to classify 333 continental sequence types into four major clusters, revealing lineage L4 as the dominant group containing 74 STs, including 13 of the 17 meat-derived isolates [175]. Such findings underscore EnteroBase’s value in identifying transmission lineages, AMR trends, and evolutionary trajectories of pathogens circulating through animal and meat-production systems.

Beyond epidemiology, EnteroBase is increasingly used as a training resource for AI and machine-learning models, due to its standardized genomic pipelines and massive curated datasets. Another study used EnteroBase core-genome MLST features to train machine-learning algorithms for automated *Salmonella* serovar prediction, achieving > 95% accuracy [176]. Similarly, another study employed EnteroBase-derived genomic features to develop a Random Forest model that predicted host association of *E. coli* isolates, demonstrating strong performance in distinguishing meat-associated lineages [177]. These studies highlight the platform’s growing relevance for AI-assisted pathogen detection and risk modeling.

### 5.4. CDC Whole-Genome Sequencing and Surveillance Programs Supporting Meat-Borne Pathogen Detection

The U.S. Centers for Disease Control and Prevention (CDC) operates one of the world’s most advanced surveillance infrastructures for foodborne and meat-associated pathogens. Since adopting whole-genome sequencing (WGS) in 2013, the CDC has transformed outbreak investigations by enabling precise strain discrimination, cluster detection, and real-time tracking of pathogens such as *Salmonella*, *E. coli*, *Campylobacter*, and *Listeria* [178,179]. The integration of WGS into routine surveillance has been particularly critical for meat-linked outbreaks; for example, CDC investigations from 2012 to 2019 documented 27 outbreaks, resulting in 1103 illnesses, 254 hospitalizations, and two deaths, with ground beef implicated in 73% of cases [180]. CDC’s Foodborne Diseases Active Surveillance Network (Food Net) and the National Antimicrobial Resistance Monitoring System (NARMS) play key roles in monitoring pathogen trends across meat and livestock systems. NARMS data have highlighted that 38% of meat-associated *Salmonella* infections involve multidrug-resistant strains, underscoring the system’s value in characterizing emerging and AMR threats relevant to meat safety [180]. These integrated datasets support epidemiological modeling, risk prediction, and evaluation of pathogen dissemination across animal, food, and human domains. The CDC datasets are increasingly used for AI model development, particularly for outbreak forecasting, anomaly detection, and genomic cluster prediction. Several recent studies have trained machine-learning models on CDC WGS and epidemiological data to predict *Salmonella* serotypes, transmission clusters, and AMR phenotypes with high accuracy. Such models rely on the CDC’s standardized genomic pipelines, making the agency a major contributor of high-quality training data for emerging AI-driven detection approaches.

### 5.5. GenomeTrakr Network and Complementary Surveillance Systems for Meat-Associated Pathogen

GenomeTrakr, maintained by the U.S. FDA, is the world’s largest publicly accessible WGS network for foodborne pathogen surveillance, integrating genomic data from food, environmental, and clinical sources to support high-resolution outbreak detection and traceback investigations [181]. Its relevance to meat safety is substantial, as many of its largest datasets, including *Salmonella*, *Listeria*, *E. coli*, and *Campylobacter*, originate from livestock, meat-processing facilities, and retail meat samples. By enabling rapid comparison of isolates against a continuously expanding global reference collection, GenomeTrakr strengthens the capacity to identify meat-associated contamination events and emerging high-risk lineages [181]. The complementary CDC-led systems PulseNet, NARMS, and FoodNet further enhance meat-borne pathogen tracking. The PulseNet’s standardized WGS and PFGE (Pulsed Field and Gel Electrophoresis) workflows enable cluster detection across different states, like the genotyping of *Enterococcus faecalis* from ground beef, which revealed that 58% of isolates formed clonal clusters, highlighting the persistence of specific meat-associated lineages in the supply chain [182]. On the other hand, the NARMS provides AMR profiles for foodborne pathogens from humans, animals, and retail meats, generating actionable insights into resistance trends affecting meat safety [183]. Additionally, the FoodNet offers incidence data and exposure patterns from surveillance sites across the United States, supporting epidemiological assessments of foodborne infections [184].

While GenomeTrakr is increasingly used as a training resource for machine-learning models that predict outbreak clusters, AMR phenotypes, and contamination sources, its broader utility is constrained by several reviewer-relevant limitations [181]. Variability in sequencing quality, inconsistent metadata (e.g., missing isolation source or processing stage), and irregular international participation hinder full cross-country comparisons. Furthermore, GenomeTrakr lacks full interoperability with other genomic platforms, such as NCBI Pathogen Detection and EnteroBase, making the integration of genomic, epidemiological, and food-chain data a challenging task [181]. These harmonization gaps limit the performance and generalizability of AI models developed for meat-borne pathogen detection. However, strengthening metadata standards, expanding global participation, and developing unified identifiers and cross-database compatibility frameworks would substantially enhance GenomeTrakr’s capacity to support next-generation molecular diagnostics and AI-driven detection pipelines for meat-associated bacterial hazards.

### 5.6. ComBase, GenBank, and BioCyc as Core Data Sources for Meat-Pathogen Characterization

ComBase, GenBank, and the BioCyc Pathway Database together provide complementary datasets that greatly enhance the study of meat-associated bacterial pathogens by supplying growth-response data, genomic information, and pathway-level functional insights. ComBase contains over 60,000 records documenting microbial behavior under diverse food environmental conditions and includes tools such as the ComBase Predictor and Food Models for estimating pathogen growth and inactivation in various food matrices [185]. Its browser allows users to examine microbial growth and survival curves generated from research studies, and the platform supports data downloads, educational use, and contributions from researchers. Moreover, case studies demonstrate their practical relevance, for example, when *Listeria* strains were grown at temperatures ranging from 2 to 35 °C, growth rates estimated using DMFit (dynamic modeling fit) were generally consistent with ComBase predictions, except for tomatoes at 30 °C and 35 °C, where ComBase showed conservative, fail-safe tendencies [186]. Similarly, when STEC growth was evaluated in ground beef under dynamic temperature cycles (5–15 °C for 300 h and 10–40 °C for 25 h), ComBase under-predicted growth at higher temperatures and failed to detect growth below 10 °C, illustrating both its utility and limitations for modeling real-world meat systems [187].GenBank, maintained by the National Center for Biotechnology Information (NCBI), serves as a global repository for publicly accessible DNA and mRNA sequences and has expanded rapidly since its launch in 1982 [188]. By December 2024 (Release 259.0), GenBank contains more than 26 trillion nucleotide bases across over 2 billion whole-genome sequences [188]. Its genomic records for meat-related pathogens, such as *L. monocytogenes* strains *F2365* (2.9 million bp) and *F6854* (2.95 million bp), have supported comparative analyses identifying 51–97 strain-specific genes and more than 100,000 SNPs related to virulence, evolutionary emergence, and diagnostic marker development [189,190]. GenBank-derived sequence information has also facilitated the design of highly sensitive PCR assays, including a *Salmonella* detection assay achieving 99.1% specificity and 100% sensitivity across 128 strains and detecting as little as 0.54 ± 0.09 log_10_ CFU/mL in beef and 1.45 ± 0.21 log_10_ CFU/mL in pork under laboratory conditions [23]. The BioCyc Pathway Database further enriches pathogen analysis by integrating curated data from over 146,000 publications and providing metabolic modeling, genome reconstruction, and comparative genomics through its Pathway Tools software. It offers extensive coverage of virulence, metabolic adaptation, and antimicrobial resistance mechanisms for major foodborne pathogens, including *Salmonella* and *Listeria*. BioCyc facilitates the analysis of genes, pathways, and metabolites, enabling researchers to explore evolutionary dynamics and survival strategies of pathogens within meat production systems, while its support for whole-genome sequencing (WGS) helps to track outbreaks and monitor AMR trends [191]. By promoting international data sharing and collaborative research, BioCyc plays a vital role in addressing the global challenge posed by emerging meat-associated bacterial threats [192]. Together, these databases provide kinetic, genomic, and metabolic datasets essential for pathogen characterization and for building robust computational frameworks to support meat safety surveillance and predictive modeling (Table 2).

**Table 2:** Summary of major genomic and bioinformatics databases relevant to meat-borne pathogens, highlighting their primary focus, analytical tools, and key applications in pathogen surveillance, virulence analysis, and antimicrobial resistance studies.

Database	Latest Version/Release	Primary Focus	Pathogen Scope	Data Type/Tools	Applications in Meat-Borne Pathogens (Updated Info)	Unique Features/Strengths	Limitations/Challenges	References
NCBI Pathogen Detection System	Real-Time (Continuous Update)	Disease & AMR (Antimicrobial Resistance) surveillance	>50 taxa (e.g., *Salmonella*, *E. coli*, *Listeria*, *Campylobacter*)	WGS (Whole Genome Sequencing), AMR FinderPlus for AMR/VF analysis, Phylogenetics	Clusters related pathogen sequences from food, environmental, and patient samples; real-time tracking of AMR genes (*mcr*, *bla*KPC, etc.)	Real-time integration; automated analysis pipeline; NDARO (National Database of AMR Organisms) linked	Metadata gaps on source/context; dependent on submitter quality and data volume	[164,166,193]
VFDB	VFDB 2025 (v6.0 is core data)	Virulence gene catalog and classification	~32 genera of well-studied bacterial pathogens (e.g., *Salmonella*, *Clostridium*, *Listeria*)	Annotated VFs, protein data, and a general classification scheme for VFs	Essential for identifying pathogenicity determinants in meat isolates; mapping VF profiles for risk assessment	Rich VF reference; distinction between core (verified) and full (predicted) datasets	Bacterial only; no direct AMR tracking; focuses on genes, not complete genomes	[40]
EnteroBase	Continuous Update (uses Hierarchical Clusters)	Global genome collection and high-resolution typing	*Salmonella*, *E. coli*/Shigella, *Vibrio*, and *Yersinia* (Enterobacteriaceae)	wgMLST/cgMLST, HierCC (Hierarchical Clustering), Assemblies, GrapeTree visualization	Provides high-resolution traceability of outbreak-associated isolates, linking source (meat, environment) to clinical cases	>1 million strains; strong genomic clustering for global traceability and source attribution	Focused taxonomy (mainly Enterobacteriaceae); data quality dependent on submitter metadata	[173,174]
CDC Databases	PulseNet 2.0 Architecture (May 2023 White Paper)	Outbreak surveillance and molecular tracking	Major foodborne & waterborne pathogens (*Salmonella*, *E. coli* O157, *Listeria*, *Campylobacter*)	WGS, Epidemiology, Public Health Reports	Integrates WGS data from clinical, food, and environmental sources to link meat-borne illnesses to the contamination source	Links genomics & epidemiology for rapid outbreak investigation; multi-national (PulseNet International)	U.S. centric reporting focus; reliance on state and federal lab reporting for completeness	[178,179,194]
GenomeTrakr Network	Continuous Update (Latest Fast Facts Aug 2025)	Foodborne pathogen & AMR tracking (Whole Genome Sequencing network)	*E. coli*, *Enterococcus*, *Salmonella*, *Listeria*, *Campylobacter*, *Vibrio*, *Cronobacter*	WGS (Sequences submitted to NCBI), AMR analysis, Epidemiology	>1.6 million isolates sequenced; rapid comparison of food and environmental isolates to human clinical isolates	Multi-agency integration (FDA, USDA, CDC, State Labs); rapid tracing and open-source data access	Potential data fragmentation across submission sites; relies on external labs for sequencing and metadata	[181,195]
ComBase	Continuous Update (Metadata Updated Dec 2025)	Predictive modeling of microbial growth and survival	*Listeria*, STEC (Shiga Toxin *E. coli*), *Salmonella*, *Clostridium* (various)	Growth & inactivation models (DMFit, Predictor, Modeling Toolbox)	Predicts pathogen behavior (growth/survival) in specific meat/food environments (e.g., temperature, pH, salt)	Real-world predictive tool for risk assessment and HACCP plan development in meat processing	Limited genomic link; models are phenotypic (not WGS-based); predictions are less reliable at extreme environmental conditions	[185]
GenBank	Release 268.0 (August 2025)	Sequence repository (Comprehensive Archive)	All organisms (including all meat-borne pathogens)	DNA/mRNA sequences, annotations, BioProject/BioSample metadata	The foundation for all genomic AI models; source for WGS data used in the other databases (e.g., NCBI-PDS)	Largest public archive (5.90 billion records, 47.01 trillion bases); high-volume, diverse data source	High redundancy and heterogeneity; reliance on manual/automated curation and annotation quality	[189,196]
BioCyc	Periodic Release (3 times per year)	Pathway & Genome Reconstruction (Functional Annotation)	>15,000 species (focused on metabolic and regulatory pathways)	Pathway Tools, Omics Visualization, Metabolic Modeling	Maps virulence and resistance pathways (e.g., quorum sensing, toxin production) in meat pathogens	146K+ curated papers; provides deep functional insight into pathogen biology	Complexity of the user interface; requires a license/subscription for full commercial use	[191]

## 6. AI-Powered Technologies in Meat Pathogen Detection, Predictive Risk Assessment

The application of AI in the detection of meat-borne pathogens is commonly characterized in terms of a given technology, like biosensors, imaging, and spectroscopy platforms. Nonetheless, this division may cause conceptual overlaps, since most of these methods are similar to computational tasks, such as classification, pattern recognition, and predictive modeling. To render a more coherent and integrative view, AI applications have been regrouped as per their functional roles, instead of the detection platforms they pertain to. This task-based framework allows for a more accurate comparison of studies and less redundancy since it underlines common computational principles underlying various technologies.

### 6.1. AI-Predictive Risk Models for Outbreak Pathogens

AI models are applied to predictive modeling, especially to evaluate the risk of contamination and trends of microbial resistance. These models combine multi-source data such as genomic sequences, environmental parameters, and historical records of outbreaks to predict contamination events, as AI-based risk modeling is not platform-dependent but cross-cutting analysis functionality. Deep learning models predict pathogen evolution and transmission routes by putting genomic sequences and transportation logs into action, such as the spread of *Salmonella* in poultry supply chains [193,197]. For instance, AI-powered imaging systems can rapidly analyze pathogen colony morphologies from culture plates [198]. Predictive AI models that integrate genomic data with epidemiological trends are also playing a pivotal role in forecasting contamination events. A prominent example includes the AI model that predicted an *E. coli* outbreak in Canada in 2021, enabling timely product recalls and reducing health risks [199]. The integration of WGS in these systems allows for precise outbreak tracing, the identification of virulence genes, and monitoring antimicrobial resistance [200]. WGS has proven vital in tracking the transmission of pathogens like *Campylobacter* in poultry and *L. monocytogenes* in processed meat products, enhancing public health response measures [201]. For example, in 2019, metagenomics helped to trace *Campylobacter* contamination in poultry in Germany, leading to stronger hygiene regulations [202]. Similarly, the 2022 outbreak of *Listeria* in Europe was traced to processed meat, which resulted in targeted recalls and more strict safety measures [203]. Reinforcement learning algorithms relate the climate-driven patterns of humidity and temperature to *Listeria* outbreaks in dairy products, allowing for recalls before the outbreak [204]. The surveillance framework is structured so that it will secure detection. Entering the first stage, AI tracks *Arcobacter* in poultry zoonotic reservoirs *via* veterinary surveillance paired with satellite imagery indicating hotspots of deforestation [176,205]. During mobility, smartphones are used to predict cross-border transmission risks like *E. coli* contamination from exported beef [206]. Outbreak detection time has been cut down from days to hours because of AI, and models like BlueDot have been able to alert with about 85% correctness during the early days of the COVID-19 pandemic [207]. AI-based pathogen detection models often face data imbalance because contaminated samples are often far fewer than non-contaminated ones, which can bias the model towards the majority class. Addressing this requires techniques like resampling, synthetic data augmentation, or cost-sensitive learning to ensure reliable detection of low-prevalence pathogens. The AI-bio-sensing framework, which applies the phage-lysis approach, achieves a 10 CFU/mL result in 5 h [208,209,210]. Additionally, convolutional neural networks (CNN) based ResNet-18 deep learning model integration into a microfluidic fluorescence digital platform has enabled direct estimation of *E. coli* from fluorescence images, achieving 99% predictive accuracy. This can have an ultra-low detection limit of 2 CFU/mL and a linear detection range spanning 10 to 3 × 10^6^ CFU/mL. When tested using the chicken matrices, the system demonstrated high recovery efficiencies (96.4–104%) and bacterial capture rates up to 100%, highlighting the strong applicability of AI-assisted bio-sensing for meat pathogen detection [211]. Moreover, for volatile organic compounds (VOCs) emitted from particular species like *L. monocytogenes*, *Salmonella*, and *E. coli* from chicken samples, the detection improvement was achieved by applying a paper chromogenic array sensor-machine learning (PCA-ML). When this was applied to real samples, the detection of Volatile organic compounds (VOCs) was achieved with an accuracy of 90% while detected CFU was as low as 1 log per gram of sample [212]. However, in another study, convolutional neural networks (CNNs) combined with surface-enhanced Raman spectroscopy (SERS) biomarkers now achieve 99.99% classification accuracy and R^2^ > 0.97 in quantitative prediction across multiple bacterial metabolites, representing a major advancement beyond classical calibration-based detection [18]. Similarly, coupling electrochemical impedance or gallium-induced (Ga-In) hydrogel systems with multilayer perceptron (MLP) models enables *E. coli* detection within 30 min with 97% predictive accuracy over wide concentration ranges (10^2^–10^9^ CFU/mL) [159]. In addition to this, machine-learning-enhanced surface-enhanced Raman scattering–based lateral flow assay SERS-LFA assays using XGBoost regression (XGBR) further allow highly quantitative detection of *E. coli 0157:H7* with an exceptional limit of detection (LOD), i.e., 6.94 × 10^1^ CFU/mL, and successful recovery at 10 CFU/mL in milk and beef ranging from 0 to 14 h [213]. Moreover, deep learning models such as Faster region-based convolutional neural networks (R-CNN) have also been utilized to enumerate fluorescently labeled *S. typhimurium*, achieving detection down to 55 CFU/mL in 2.5 h, while ML-assisted Raman volatile organic compounds (VOC) fingerprinting enables accurate, real-time classification of *E. coli*, *Salmonella*, and *Listeria* signatures even at 100× dilutions. Additionally, random forest classifiers integrated with cell-imprinted electrochemical sensors to allow qualitative identification and semi-quantitative measurement of *E. coli*, *S. aureus*, and *V. parahaemolyticus* across 10^1^–10^6^ CFU/mL, offering low-cost and field-ready diagnostics [214]. Many reported models are trained on controlled or limited datasets that may not fully represent real-world variability across food matrices and pathogen strains. In several studies, model performance is evaluated using internal cross-validation rather than independent external datasets, which can inflate predictive accuracy and limit the assessment of model robustness across geographically diverse pathogen populations. Larger and multi-source datasets are necessary to improve model generalization and field applicability. Research incorporating ML tools alongside Raman spectroscopy can detect seven pathogen genera: *Escherichia*, *Listeria*, *Staphylococcus*, *Cronobacter*, *Vibrio*, *Shigella*, and *Salmonella* [211]. Two ML classification algorithms, K-PCA DT (K-Principal Component Analysis Decision Tree) and PCA-SVM (Principal Component Analysis Support Vector Machine), were tested, which successfully classified and detected pathogens with accuracies ranging from 87% to 96%, with K-PCA DT exhibiting superior discrimination performance compared to PCA-SVM. Moreover, in the four-level classification models of K-PCA DT, the prediction accuracy at the top level (92.2%) was higher than at the genus (88.6%), species (88.3%), and serotype (88.4%) levels [108,210]. The classification accuracy followed the order *Escherichia* > *Listeria* > *Staphylococcus* > *Cronobacter* > *Vibrio* > *Shigella* > *Salmonella*, although it varied across different strains. It is also reported that surface-enhanced Raman spectroscopy (SERS), when combined with a machine learning (ML) tool, can identify methicillin-resistant *Staphylococcus aureus* (MRSA) bacteria. In SERS, active surfaces are brought into proximity with molecules, enhancing scattered signal intensity through the surface plasma effect. This study utilized 10 MRSA, three methicillin-sensitive *Staphylococcus aureus* (MSSA), and 6 *Legionella pneumophila* isolates [215]. The Raman spectra were analyzed using supervised ML algorithms, including SVM, K-nearest neighbors (KNN), decision trees (DT), and naive Bayes, as well as unsupervised methods such as principal component analysis (PCA) and hierarchical cluster analysis (HCA) [216]. Results indicated that the supervised KNN algorithm outperformed the others, achieving 97.8% accuracy compared to SVM (92.3%), DT (88.9%), and naive Bayes (82.8%). Additionally, PCA demonstrated superior classification performance over HCA [217]. Deep learning classifiers often function as “black boxes”, making it difficult to interpret the type of spectral features, genomics markers, or environmental factors that influence prediction. Furthermore, incorporating explainable AI approaches can enhance transparency, regulatory acceptance, and user trust.

**Figure 4:**
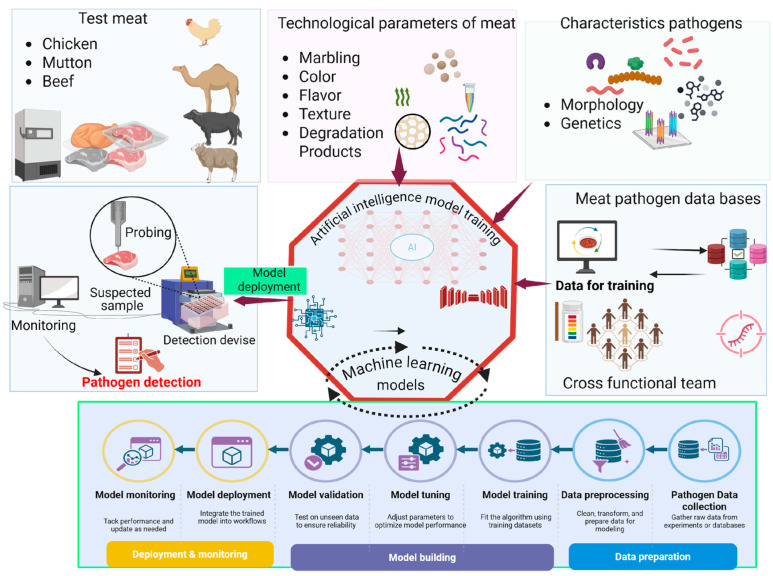
Overview of the AI-enabled workflow for meat-borne pathogen detection. The figure illustrates how meat samples, technological meat parameters, pathogen characteristics, and curated pathogen databases are integrated to train machine-learning models for contamination detection. AI models are developed through data collection, preprocessing, training, tuning, and validation, then deployed into detection devices for real-time monitoring and continual performance updates.

Another study mapped and predicted the spatiotemporal patterns of *salmonellosis* in Northwestern Italy, utilizing confirmed human cases from 2015 to 2018 (*n* = 1969) and food surveillance data from January 2014 to December 2018 to train machine learning (ML) models. The random forest and gradient boosting models achieved R-squared values of 0.55 and 7.5% MAPE (mean absolute percentage error), respectively, while the tree regression algorithm obtained R-squared and MAPE values of 0.42 and 8.8%, respectively. Key factors enhancing model prediction capacity included *Salmonella* prevalence in food, spatial characteristics, and monitoring of ready-to-eat dairy products, fruits, vegetables, and pig meat, thereby reducing variants by 90.5%. In contrast, the number of positive samples from specific food matrices had a negligible effect on predictions (2.9%) [218] (Figure 4). Deep learning models have achieved pathogen classification accuracies exceeding 97% for pathogens like *Salmonella* and *E. coli* [193,219]. AI-driven approaches, ranging from deep learning classifiers and bio-sensing platforms to WGS-integrated surveillance systems, have transformed the speed, accuracy, and resolution of food-borne pathogen detection across the entire farm-to-fork continuum. These innovations demonstrate that AI can predict outbreaks earlier, trace contamination events with high precision, and enable ultra-sensitive, real-time detection of pathogens in complex food matrices, ultimately strengthening public health response and food safety management. Despite these high predictive accuracies, most AI-based outbreak models remain decision-support tools rather than standalone diagnostic replacements, as their performance is highly dependent on historical data distributions that may shift over time due to changes in processing practices, climate conditions, or pathogen evolution [220]. Such data drift can substantially degrade model reliability in real-world deployment and necessitates continuous and consistent external validation. In food safety monitoring systems, data drift may occur when microbial populations evolve, processing conditions change, or sensor performance varies between facilities, thereby altering the statistical distribution of the data relative to the original model training environment. Moreover, many deep learning–based outbreak prediction systems operate as black-box models, which limits their interpretability because they operate based on genomic, environmental, or supply-chain variables for risk predictions [220]. This lack of transparency remains a significant barrier to regulatory acceptance, particularly when AI outputs conflict with conventional microbiological findings.

### 6.2. AI-Integrated Biosensing and Electrochemical Systems

Al-integrated biosensing and electrochemical systems represent some of the most rapid and sensitive pathogen-detection strategies currently emerging for meat safety assessment. These platforms combine biological recognition elements (such as bacteriophage-lysis sensors, immuno-biosensors, or impedance-based electrodes) with machine learning (ML) algorithms that refine signal interpretation and significantly enhance analytical precision [221,222]. Al-biosensing frameworks typically detect pathogens such as *Salmonella* and *E. coli* directly from meat juices within 4–5 h, achieving extremely low detection limits near 10 CFU/mL, with model accuracies approaching ˜99% after training. Their major advantage lies in automated readouts and reduced operator bias, although they require periodic ML model recalibration to maintain performance [208]. Electrochemical Al-enhanced sensors, in contrast, provide much faster detection, typically within 10–40 min, by interpreting changes in impedance or voltammetry signals from contaminated meat matrices. ML models such as ANN or SVM improve the signal-to-noise ratio and push detection limits to 10–100 CFU/mL, with accuracies ranging between 92 and 97%, depending on fat content or marbling, electrode type, and sample preparation quality [223]. Although these are slightly less sensitive than biosensing systems, their field applicability, low cost, and ability to process complex meat matrices in real time provide clear operational benefits. However, electrode fouling in high-fat meat samples remains a key limitation. Microfluidic Al-assisted biosensing platforms form the third pillar of this group. These systems integrate chip-based bacterial capture technologies with ML-guided decision models, enabling detection from meat homogenates in 20–60 min and at sensitivity levels as low as 1–50 CFU/mL. These devices outperform electrochemical sensors in raw sensitivity while maintaining a considerably faster turnaround than classical biosensing frameworks [224]. Nevertheless, issues such as chip clogging due to fat and protein particulates restrict their performance in real-world slaughterhouse environments. Despite these challenges, AI-integrated biosensing, microfluidics, and electrochemical sensors collectively demonstrate high detection precision (92–99%), low sample-load thresholds, and improved robustness compared with traditional analytical methods, positioning them as highly promising front-line tools for rapid meat-borne pathogen monitoring [225].

Al-integrated biosensing platforms represent one of the most transformative advancements in rapid pathogen detection, especially when combined with imaging, microfluidics, phage-based assays, and smartphone-enabled analytics. A notable development is the computational live-bacteria detection system incorporating time-lapse coherent imaging and deep learning (DL) for ultra-fast microbial growth analysis. This system detects and classifies *E. coli*, *K. aerogenes*, and *K. pneumoniae* with 90% detection accuracy and 80% classification accuracy, while reducing total detection time by >12 h compared to EPA-approved gold-standard methods. Importantly, it reaches an exceptional limit of detection (LOD) of 1 CFU/L within 9 h, making it significantly more sensitive than most conventional plate-based assays. Its low-cost design enables integration with agar plates and other standard bacterial testing workflows. For example, smartphone-enabled paper microfluidic assays coupled with supervised machine learning (SVM) achieved 93.3% accuracy in bacterial species classification. In this system, peptide-conjugated particles interact with bacterial suspensions, and the resulting flow-velocity fingerprints are interpreted by ML models to differentiate species, demonstrating an accessible, portable screening tool with high diagnostic 4′ power (power probe 4) [221]. Another promising advancement is an AI-driven biosensing framework using bacteriophage–bacteria interaction signatures analyzed through DL to detect pathogens in agricultural water and liquid food matrices. This system produces quantitative predictions within 5.5 h and demonstrates 80–100% accuracy when tested on real-world water samples containing diverse background contaminants. By automating microscopic pattern interpretation and eliminating the need for extensive human expertise, this platform significantly reduces labor, operational complexity, and turnaround time in environmental and food-safety monitoring [226]. Collectively, these Al-integrated biosensing and electrochemical approaches provide high sensitivity (down to single-cell LOD), rapid detection (<1–6 h in many systems), and strong model performance (>90% accuracy), substantially outperforming conventional culture-based and biochemical methods. Additionally, most reported performance metrics are obtained using artificially inoculated samples under controlled conditions, whereas naturally contaminated meat contains heterogeneous microbiota, debris, and physicochemical variability that can significantly impair sensor performance and model robustness. However, the translation of these biosensing platforms from laboratory settings to industrial meat processing environments remains limited. High fat and protein content in meat matrices frequently causes electrode fouling, signal instability, and microfluidic channel clogging, leading to reduced reproducibility and increased maintenance requirements compared with culture-based assays [227,228].

### 6.3. AI-VOC/E-Nose Technologies

Artificial intelligence–enhanced electronic-nose (E-nose) systems are emerging as powerful tools for the rapid, non-invasive detection of microbial contamination in food products. These platforms analyze volatile organic compound (VOC) signatures released by bacteria during growth and employ machine learning models to interpret complex odor profiles with high precision [229]. By integrating gas-sensor arrays, chemometric features, and AI-driven pattern-recognition algorithms, AI-E-nose technologies enable early identification of spoilage and pathogenic bacteria, offering faster, cost-effective, and field-deployable alternatives to traditional microbiological assays. To detect volatile organic compounds (VOCs) emitted by foodborne pathogens, Yang et al. created a paper-based chromogenic array impregnated with 23 dyes. The resulting complicated colorimetric patterns were processed with 91–95% accuracy using a multilayer neural network to identify *E. coli*, *L. monocytogenes*, and *S. aureus* in fresh-cut lettuce that had been subjected to temperature abuse. Without the need for enrichment or incubation, a deep feed-forward neural network in conjunction with a paper-based colorimetric array also made it possible to detect pathogen-specific volatile organic compounds (VOCs) in shredded cheddar cheese, attaining 85–92% accuracy at 3–5 log CFU/g and 72–96% at 1 log CFU/g within 24 h. Similarly, *L. monocytogenes*, *S. enterica*, and *E. coli O157:H7* were detected in ground chicken using chromogenic dye interactions with volatile organic compounds (VOCs). The system maintained greater than 80% accuracy within 24 h at 4 °C and more than 90% accuracy at contamination levels as low as 1 log CFU/g within 5–7 h at 25 °C [230]. Similarly, Cui et al. improved the field by developing an AI-assisted smartphone-based colorimetric biosensor that targets hyaluronidase, an enzyme released during bacterial infection. The system used a hydrogel-based bioreactor and signal generator, where color changes caused by enzyme-mediated degradation were examined using a YOLOv5 algorithm on a smartphone interface. This platform effectively differentiates between Gram-positive and Gram-negative bacteria, as well as between live and dead cells, with an ultra-low detection limit of 10 CFU/mL in 60 min. This system performance was validated in blueberries, where antimicrobial susceptibility testing and clinical specimens were also used, yielding 100% sensitivity and specificity values [231]. With ≥80–100% accuracy across a variety of food matrices and contamination levels, AI-enhanced VOC/E-nose technologies show great promise for quick, non-invasive foodborne pathogen identification without the need for enrichment or complicated laboratory procedures. Although AI-enhanced VOC and E-nose systems demonstrate strong classification performance, their broader industrial adoption is constrained by sensor drift, batch-to-batch variability, and limited odor reference libraries. These factors can cause progressive degradation in model accuracy over time, requiring frequent recalibrations in continuous processing environments [228]. Furthermore, VOC profiles are strongly influenced by storage conditions, packaging materials, background spoilage flora, and environmental humidity, which complicates model generalization across facilities and limits the reliability of these systems as confirmatory diagnostic tools.

### 6.4. AI-Vision, Imaging, and Spectroscopy Methods

One of the most effective methods for quickly and non-destructively identifying foodborne pathogens in meat and poultry systems is artificial intelligence-driven visual analytics. These technologies offer high-resolution spatial and spectral mapping of microbial contamination by combining computer vision, deep learning, hyperspectral imaging (HSI), and infrared (IR) sensing that is not possible with classic microbiological plating techniques [232]. Because of this characteristic, CNNs are better at extracting spatial characteristics from photos related to food than conventional fully connected neural networks (FCNNs). However, while processing the food photos, the convolution operation of CNNs enables the networks to detect local characteristics in a translation-invariant manner, regardless of where they occur in the image. This is crucial because contaminants or flaws can appear in any region of the image. Convolutional neural networks (CNNs) in particular are capable of autonomously classifying bacterial colonies, identifying contamination patterns, and deciphering complex spectral signatures with accuracy levels over 95% [233,234]. The potential of hyperspectral imaging integration with AI is evident from numerous studies owing to its sensitivity and non-destructive food safety monitoring. For instance, using a dataset of 210 uncontaminated and 210 contaminated images, a short-wave infrared HSI system in conjunction with 1D-CNN, PLS-DA, and SVM successfully identified contamination status and pathogen types in mutton, achieving 92.86% accuracy on the test set and 97.62% accuracy on an external validation set [235]. With remarkable robustness, a different deep CNN-based electromechanical platform with a time-of-flight sensor identified nine distinct packaging contaminants in 2700 RGB photos with 99.74% accuracy [236]. Moreover, real-time foreign contamination identification was reported by using hyperspectral cameras and CNN models with 94% accuracy across 210 tray photos, encompassing both contaminated and normal samples, in package integrity studies [42]. Furthermore, *Clostridium* and *Bacillus cereus* spores were detected and quantified using 4250 hyperspectral pictures at different concentrations utilizing HIS in conjunction with 1D-CNN and Random Forest algorithms, yielding 90–94% accuracy. When taken as a whole, these results demonstrate the robust diagnostic capabilities, dependability, and flexibility of HSI-AI platforms for various food contamination situations [236]. In one study, spectral images from 100 samples were used to evaluate the fat content of salmon filets using near-infrared hyperspectral imaging (NIR-HSI) combined with a Residual Attention Convolutional Neural Network (RACNN). In a controlled laboratory setting, the optimized predictive model demonstrated good predictive capabilities with a performance coefficient of determination (R^2^_p_ = 0.90). Similarly, based on spectral datasets of 252 samples, linoleic acid concentration in red meat was measured using hyperspectral imaging in conjunction with a CNN–Bi-LSTM (Convolutional Neural Network–Bidirectional Long Short-Term Memory) hybrid model [237]. Nonetheless, the remarkable R^2^_p_ = 0.91 reported by this dual-stage model demonstrates its ability to capture nonlinear spectral–chemical connections. When taken as a whole, current research shows AI-enhanced hyperspectral systems are becoming reliable for non-destructive biochemical evaluation of animal products. Hence, future research must concentrate on enhancing real-world transferability, lowering model complexity, and validating these systems under various industrial circumstances, even in spite of their strong laboratory performance. Furthermore, harnessing AI with optical imaging offers a promising tool to detect and classify microbial load in diverse food matrices, as this approach can detect an array of pathogenic bacterial species. However, antimicrobial resistance profiling and data mining for bacterial strains by applying ML in various chicken farms and slaughterhouses offer excellent surveillance for healthy livestock production [163] (Figure 4, Table 3). Despite the consistently high accuracies reported for AI-driven imaging and hyperspectral systems, most models are trained on relatively small, curated datasets that may not capture the full variability of industrial meat processing conditions, including lighting fluctuations, surface heterogeneity, and cross-contamination patterns. As a result, model transferability between laboratories, slaughterhouses, and geographic regions remains a critical challenge, and extensive site-specific calibration is often required [228,238]. These constraints have limited the replacement of traditional culture-based methods, which, although slower, yet provide standardized, legally defensible results across diverse operational settings.

The AI-based detection systems also improve the health outcomes of the population due to the possibility of monitoring the population and giving early warnings. Using the combination of genomic, environmental, and epidemiological data, AI models will be able to detect the new contamination patterns and predict the possible outbreaks prior to their occurrence. This initiative-taking measure enables the regulatory bodies and other stakeholders in the food industry to take preventive measures, which will decrease the chances of the prevalent diseases. The success of these systems, however, requires the presence of high-quality and representative datasets and solid validation plans, which continue to be major obstacles to their extensive use in practical health settings.

### 6.5. Methodological Considerations in AI-Based Pathogen Detection

Although AI-based pathogen detection systems showed high output, many methodological factors that are extremely critical in determining their reliability and generalizability are involved [239]. Supervised algorithms like support vector machines (SVM), random forests (RF), k-nearest neighbors (KNN), and gradient boosting, and deep learning models like convolutional neural networks (CNNs), ResNet variants, and recurrent neural networks (RNNs) are commonly applied machine learning models in spatiotemporal prediction [240,241,242,243]. Normalization, noise filtering, dimensionality reduction (e.g., principal component analysis, PCA), and feature extraction of spectral, genomic, or imaging data are common data preprocessing steps [244,245,246]. Moreover, preprocessing, including baseline correction, smoothing, and wavelength selection, is a crucial step in hyperspectral and Raman-based studies to enhance signal quality and model strength [247]. The strategy of validation of models in numerous studies often uses k-fold cross-validation, train-test splits, and rarely independent external validation datasets [247,248]. The sparse application of external validation is a significant weakness, with internal validation being the sole method that can overestimate the model performance [249]. Also, the issue of class imbalances can be resolved with the help of resampling methods like SMOTE (synthetic minority over-sampling technique) or cost-sensitive learning methods to enhance the detection of low-prevalence pathogens.

On the deployment side, there are a number of challenges, such as model drift because of changing microbial populations, food matrix variability, sensor variability, and regulatory frameworks that are not yet standardized to apply AI-based diagnostics [250]. Moreover, a lot of models need regular retraining and calibration to be able to perform in various environmental and industrial conditions. These methodological and translational challenges need to be addressed to successfully incorporate AI into the normal food safety monitoring mechanisms.

### 6.6. Overall Assessment of AI-Based Detection Systems

Even though artificial intelligence has become a formidable solution in the process of detecting pathogens, its real-world applications in meat safety systems are in a translational stage [251]. Most of the studies indicate high classification accuracies, such as above 95% in many cases, especially in scenarios like serovar determination of Salmonella, but these findings are often achieved by using small databases that are produced in controlled laboratory settings [252]. These conditions are not sufficient owing to variability in a real-world meat processing environment, such as sample composition, contamination, and environmental noise variability [253]. The main limitation of the existing AI-based systems is overfitting, when the model is effective on the training data but cannot handle unseen datasets [254]. Moreover, imbalance in datasets and unverified external validation are other elements that undermine the reliability of reported performance metrics [255].

Another important limitation of AI-based detection systems is the issue of dataset bias. This problem arises due to the usage of non-diverse datasets in several research studies. For instance, imaging datasets might have been collected using a consistent light source and in controlled lab settings, whereas genomic datasets could be biased towards certain strains or geographical locations. The issue of dataset bias results in a reduced generalizability capacity of the developed AI models in real-world meat processing facilities with high variability in temperatures and contamination rates. Furthermore, the domain shift, which refers to the distinction between training samples and real-world samples, is one of the major obstacles faced by artificial intelligence technology. Nonetheless, differences in sample quality, background noise, sensor tuning, and processing environment could result in poor performance. In an industrial setting, there are more complications such as data diversity, scarce data labeling, and incorporation with food safety protocols.

In a methodological sense, convolutional neural networks (CNNs) are most often utilized in imaging and hyperspectral analysis because they are capable of identifying both spatial and spectral features, whereas machine learning models like Random Forest and Support Vector Machines are often used in genomic and biosensor data analysis because they are less sensitive to structured data. Nevertheless, data availability usually influences the choice of models but not biological relevance, as it demonstrates a disparity between computational optimization and practical applicability. Moreover, most of the research highlights accuracy as the major measure of evolution, whereas it overlooks critical performance measures like sensitivity, specificity, precision, recall, and model calibrations. These restrain the interpretability and regularity acceptability of AI-based systems to be used in food safety. Thus, in future studies, emphasis should be placed on solutions to make sure that such systems can be reliably used in industrial meat processing and in community health surveillance.

**Table 3:** AI-enabled detection approaches for meat-borne bacterial pathogens. The table compares major AI–assisted methods used in meat safety diagnostics, outlining their detection principles, performance metrics, on-site applicability, limitations, and added benefits over traditional techniques.

Category/Detection Approach	Principle/Example (Meat Context)	Detection Time	Sensitivity/Detection Limit	Accuracy/Specificity	Cost/Test ($)	Field/On-Site Applicability	Major Limitations	AI-Added Benefit	References
AI-Biosensing Frameworks	Phage-lysis or immuno-biosensors + ML signal analysis for *Salmonella*/*E. coli* in meat juices	4–5 h	10 CFU/mL	~99%	$2–10	Yes	Requires ML model calibration	Higher sensitivity; automated readout	[208]
AI-Vision & Deep Learning Detection	CNNs classify colony morphology from meat rinse plates or hyperspectral images	1–3 h	≤10 CFU/mL	97–99%	$1–5	Fully automated	Needs large annotated datasets	Rapid auto-detection	[256]
AI + Spectroscopy (ML–SERS)	ML interprets Raman/SERS spectra for *Listeria* and *E. coli* in raw meat	<1 h	10^3^ CFU/mL	94–98%	$5–20	Yes	Spectral overlap; retraining needed	Improves spectral discrimination	[257]
AI Predictive & Risk Models	Deep learning predicts *Salmonella* growth in poultry/beef based on temperature & handling	Minutes–hours	NA	85–95% predictive accuracy	$0.1–1	Yes	Data bias; integration challenge	Early contamination prediction	[258]
AI-Spatiotemporal Mapping	ML predicts slaughterhouse/processing “hotspots” of *Salmonella*	Real-time	NA	R^2^ = 0.55; MAPE = 7.5%	$0.5–2	Yes	Hard to detect hidden clusters	High-risk area identification	[259]
AI-Enhanced qPCR/RT-qPCR	ML improves Ct analysis for *E. coli* O157:H7, *Listeria*, and *Campylobacter* in meat	30–90 min	1–10 copies/reaction	>99%	$20–60	Limited	Needs DNA extraction	Reduces false positives/negatives	[260]
AI-Microfluidic Lab-on-Chip Detection	ML-guided microfluidic capture of bacteria from meat rinse or homogenates	20–60 min	1–50 CFU/mL	95–99%	$10–40	Yes	Chip clogging from fat/protein	Automated pathogen separation	[261]
AI-Electrochemical Sensors	ANN/SVM interprets impedance/voltammetry signals of *Salmonella*/*E. coli* in meat juices	10–40 min	10–100 CFU/mL	92–97%	$3–15	Yes	Electrode fouling in high-fat meats	Noise reduction & better detection	[231,262]
AI-VOC/E-Nose for Meat Bacteria	ML differentiates VOC profiles of meat contaminated with *Pseudomonas*, *Salmonella*, *S. aureus*	5–20 min	10^2^–10^3^ CFU/mL	88–96%	$2–10	Yes	VOC overlap; not species-specific	Early spoilage & contamination indication	[158]
AI-Infrared/Hyperspectral Imaging of Contaminated Meat	Deep learning detects bacterial contamination zones in poultry/beef via IR/HSI	1–5 min	Indirect	90–96%	$0.5–3	Yes	Indirect; needs calibration	Rapid non-destructive screening	[143]
AI-Metagenomic Read Classification	Deep neural classifiers (e.g., Kraken2-DL) detect pathogens from meat shotgun sequencing	6–12 h	~1% relative abundance	95–99%	$100–500	No	Expensive sequencing	Detects multiple pathogens simultaneously	[154]

### 6.7. Pathogen Detection Technology in the Meat Industry

Pathogen detection technology in the meat industry will depend not only on accuracy and reliability but also on feasibility within industrial settings [263]. The key aspects that determine the feasibility of technology include scalability, cost-effectiveness, difficulty in performing, skill requirements of the personnel involved, and adherence to regulatory standards [264].

Traditional culture methods provide an effective, accurate approach to detecting pathogens; however, they can take too long due to their manual nature and may be unsuitable for high-volume applications [265,266]. However, PCR and other molecular methods provide extremely sensitive and specific detection of bacteria. Nevertheless, such approaches require specialized laboratory equipment and specially trained personnel. This might prove problematic for small and medium enterprises in the meat-processing industry [267,268]. Isothermal amplification techniques represent a viable approach in this respect because they are less dependent on equipment, and the processing time is shorter than other approaches, thus better suited to field applications [269]. The biosensor approach has its advantages, such as high portability, fast detection speed, and the possibility of continuous measurement. The biosensors have much promise as part of the production line, but there are problems with sensor reliability and calibration that need to be resolved first [270]. Detection technologies using integrated artificial intelligence add yet another level of scalability through automated data analytics, high-throughput screening, and surveillance capabilities [271]. Nevertheless, such solutions require sophisticated data infrastructure, computing power, and validated algorithms that are not yet available in industrial environments [272]. Speaking of regulation, current practices involving conventional and PCR approaches have proved to be dependable because of standardization and performance confirmation [273]. New detection technologies based on biosensors and AI solutions still lack validation and face difficulties getting regulatory approval due to this reason [274]. In summary, the applicability of technologies in detecting pathogens is determined by both technological performance and business-related aspects. Approaches combining high-speed screening and verification tests are expected to yield more feasible results.

## 7. Future Directions: Regulatory, Cost, Training, Ethical, and Data Privacy Challenges

Future advancement in meat-borne pathogen detection depends not only on new diagnostic technologies but also on their ability to address the interconnected regulatory, economic, technical, ethical, and data-governance challenges that currently limit their widespread application. Based on the current review, readers can equip themselves with a number of tools, techniques, comparative applications, and future goals. Although artificial intelligence integration with biosensors, nanomaterial-based assays, CRISPR-Cas platforms, whole-genome sequencing, metagenomics, and advanced imaging has greatly improved detection speed, sensitivity, and on-site feasibility, the performance of these systems remains constrained by the quality and consistency of the underlying datasets. Many public genomic resources still suffer from incomplete metadata, inconsistent annotation standards, and substantial geographic bias, with isolates from high-income regions dominating global repositories. As a result, AI models trained on these imbalanced datasets may misclassify or underperform when confronted with emerging pathogens that circulate primarily in low- and middle-income countries, creating inequities in detection accuracy and outbreak response. Hence, modern tools, applications, and futuristic innovations demand a comprehensive framework to avoid the above-stated challenges. The economic realities of deploying advanced molecular diagnostics further widen the gap between large and small processors, since the costs associated with sequencing platforms, microfluidic devices, biosensor fabrication, computational infrastructure, and continuous personnel training remain out of reach for many facilities. At the same time, modern laboratories require cross-disciplinary expertise that is still scarce, as effective interpretation of WGS outputs, AMR gene profiles, and machine-learning predictions demands strong bioinformatics competencies alongside traditional microbiological skills. Ethical and biosecurity considerations are needed for high-resolution genomic surveillance, supply-chain vulnerabilities, unrestricted sharing of pathogen genomes, and metadata-related concerns about privacy, commercial confidentiality, and potential misuse. Hence, to translate technological advances into routine practice, coordinated global efforts are needed to improve data standardization, develop interpretable and bias-aware AI models, create affordable and scalable diagnostic platforms, expand cross-disciplinary training programs, and establish secure, ethically grounded frameworks. Only such an integrated, futuristic outlook can produce next-generation detection systems that will be mature, reliable, equitable, and globally coordinated.

## 8. Conclusions

Urbanization and industrialization have provided us with many conveniences, but the careless use of resources has led to various diseases and environmental problems. Previously, numerous studies have indicated meat-borne pathogen detection methods, but data regarding meat matrices, pathogen types, AI models, and the link between conventional and modern methods is scarce. Current review offers a number of options and decision supports instead of confining to a single tool or technique. Conclusively, the application of AI-based tools for microbial detection in abattoirs, meat processing plants, transportation, and storage facilities demands prospective validation, regulatory acceptance, and benchmarking, requiring full deployment or readiness. Among these, foodborne pathogens in meat are particularly concerning for the large population that consumes meat. Traditional and modern techniques for detecting bacterial pathogens have significantly controlled these threats. While many bacterial groups are known to cause foodborne illnesses, i.e., *Salmonella*, *Listeria*, *E. coli*, and *Shigella,* are among the most significant contributors to the global burden of disease (GBD). Numerous countries have analyzed raw and processed meat samples and identified microbes that cause food poisoning, produce bacteriotoxins, and lead to infections. Although various control strategies are employed to combat foodborne illnesses, bioinformatics and genomics have proven especially valuable for identifying microbial drug resistance, exploring phylogenetic relationships, and understanding the pathways by which these pathogens contaminate the food, particularly animal-origin products. Currently, as scientists are discovering new microbial strains and subtypes every day, a systematic approach is required. This not only integrates online databases with laboratory tools but also facilitates faster progress, streamlining workflows, and accelerates the discovery of solutions for foodborne illnesses. In conclusion, the current review highlights the significance of novel detection methods and tools for the timely diagnosis of food contamination by various bacterial strains, as conventional methods are labor-intensive and time-consuming.

## Data Availability

All data used in the draft are provided.

## References

[1] Bhat R., Jõudu I. (2019). Emerging issues and challenges in agri-food supply chain. Sustain. Food Supply Chain.

[2] Gao R., Liu X., Xiong Z., Wang G., Ai L. (2024). Research progress on detection of foodborne pathogens: The more rapid and accurate answer to food safety. Food Res. Int..

[3] Grace D. (2015). Food safety in low and middle income countries. Int. J. Environ. Res. Public Health.

[4] Almashhadany D.A. (2021). Meat borne diseases. Meat and Nutrition.

[5] Pobiner B. (2016). Meat-eating among the earliest humans. Am. Sci..

[6] Khara T., Riedy C., Ruby M.B. (2021). A cross cultural meat paradox: A qualitative study of Australia and India. Appetite.

[7] Miller V., Reedy J., Cudhea F., Zhang J., Shi P., Erndt-Marino J., Coates J., Micha R., Webb P., Mozaffarian D. (2022). Global, regional, and national consumption of animal-source foods between 1990 and 2018: Findings from the Global Dietary Database. Lancet Planet. Health.

[8] Mor-Mur M., Yuste J. (2010). Emerging bacterial pathogens in meat and poultry: An overview. Food Bioprocess Technol..

[9] Petrović T., D’Agostino M. (2016). Viral contamination of food. Antimicrobial Food Packaging.

[10] Velebit B., Radin D., Teodorovic V. (2015). Transmission of common foodborne viruses by meat products. Procedia Food Sci..

[11] Ali S., Alsayeqh A.F. (2022). Review of major meat-borne zoonotic bacterial pathogens. Front. Public Health.

[12] Omer M.K., Álvarez-Ordoñez A., Prieto M., Skjerve E., Asehun T., Alvseike O.A. (2018). A systematic review of bacterial foodborne outbreaks related to red meat and meat products. Foodborne Pathog. Dis..

[13] Gómez I., Janardhanan R., Ibañez F.C., Beriain M.J. (2020). The effects of processing and preservation technologies on meat quality: Sensory and nutritional aspects. Foods.

[14] Geiker N.R.W., Bertram H.C., Mejborn H., Dragsted L.O., Kristensen L., Carrascal J.R., Bügel S., Astrup A. (2021). Meat and human health—Current knowledge and research gaps. Foods.

[15] Mohammed M.A., Sallam K.I., Tamura T. (2015). Prevalence, identification and molecular characterization of Cronobacter sakazakii isolated from retail meat products. Food Control.

[16] Liu Q., Jin X., Cheng J., Zhou H., Zhang Y., Dai Y. (2023). Advances in the application of molecular diagnostic techniques for the detection of infectious disease pathogens. Mol. Med. Rep..

[17] Kreitmann L., Miglietta L., Xu K., Malpartida-Cardenas K., D’Souza G., Kaforou M., Brengel-Pesce K., Drazek L., Holmes A., Rodriguez-Manzano J. (2023). Next-generation molecular diagnostics: Leveraging digital technologies to enhance multiplexing in real-time PCR. TrAC Trends Anal. Chem..

[18] Kumar A., Islam M.R., Zughaier S.M., Chen X., Zhao Y. (2024). Precision classification and quantitative analysis of bacteria biomarkers via surface-enhanced Raman spectroscopy and machine learning. Spectrochim. Acta Part A Mol. Biomol. Spectrosc..

[19] Chen Q., Wu D., Yang Z., Sun C., Tang S., Chen C., Wei B., Liu Q., Bai P., Zhang H. (2025). Isolation, Identification, Antimicrobial Resistance Testing, and Whole-Genome Sequencing Analysis of Pathogenic Bacteria Causing Yak Calves Diarrhea in Qinghai Province. Res. Sq..

[20] Wu L., Li G., Xu X., Zhu L., Huang R., Chen X. (2019). Application of nano-ELISA in food analysis: Recent advances and challenges. TrAC Trends Anal. Chem..

[21] Di Febo T., Schirone M., Visciano P., Portanti O., Armillotta G., Persiani T., Di Giannatale E., Tittarelli M., Luciani M. (2019). Development of a capture ELISA for rapid detection of Salmonella enterica in food samples. Food Anal. Methods.

[22] Ohk S.-H., Bhunia A.K. (2013). Multiplex fiber optic biosensor for detection of Listeria monocytogenes, Escherichia coli O157: H7 and Salmonella enterica from ready-to-eat meat samples. Food Microbiol..

[23] Lee S.H., Jung B.Y., Rayamahji N., Lee H.S., Jeon W.J., Choi K.S., Kweon C.H., Yoo H.S. (2009). A multiplex real-time PCR for differential detection and quantification of *Salmonella* spp., Salmonella enterica serovar Typhimurium and Enteritidis in meats. J. Veter Sci..

[24] Alves J., Marques V.V., Pereira L.F.P., Hirooka E.Y., DE Oliveira T.C.R.M. (2012). Multiplex PCR for the detection of *Campylobacter* spp. and *Salmonella* spp. in chicken meat. J. Food Saf..

[25] Rajalakshmi S. (2017). Different types of pcr techniques and its applications. Int. J. Pharm. Chem. Biol. Sci..

[26] Cook N. (2003). The use of NASBA for the detection of microbial pathogens in food and environmental samples. J. Microbiol. Methods.

[27] Vu Q.K., Tran Q.H., Vu N.P., Anh T.-L., Le Dang T.T., Matteo T., Nguyen T.H.H. (2021). A label-free electrochemical biosensor based on screen-printed electrodes modified with gold nanoparticles for quick detection of bacterial pathogens. Mater. Today Commun..

[28] Hu Q., Wang S., Duan H., Liu Y. (2021). A fluorescent biosensor for sensitive detection of Salmonella typhimurium using low-gradient magnetic field and deep learning via faster region-based convolutional neural network. Biosensors.

[29] Davis D., Guo X., Musavi L., Lin C.-S., Chen S.-H., Wu V.C. (2013). Gold nanoparticle-modified carbon electrode biosensor for the detection of Listeria monocytogenes. Ind. Biotechnol..

[30] Rai M., Bonde S., Yadav A., Plekhanova Y., Reshetilov A., Gupta I., Golińska P., Pandit R., Ingle A.P. (2022). Nanotechnology-based promising strategies for the management of COVID-19: Current development and constraints. Expert Rev. Anti. Infect. Ther..

[31] Pandit S., Dasgupta D., Dewan N., Prince A. (2016). Nanotechnology based biosensors and its application. Pharma Innov..

[32] Banerjee A., Maity S., Mastrangelo C.H. (2021). Nanotechnology for biosensors: A Review. arXiv.

[33] Sun X., Wang Y., Zhang L., Liu S., Zhang M., Wang J., Ning B., Peng Y., He J., Hu Y. (2020). CRISPR-Cas9 triggered two-step isothermal amplification method for *E. coli* O157: H7 detection based on a metal–organic framework platform. Anal. Chem..

[34] Makarova K.S., Wolf Y.I., Alkhnbashi O.S., Costa F., Shah S.A., Saunders S.J., Barrangou R., Brouns S.J., Charpentier E., Haft D.H. (2015). An updated evolutionary classification of CRISPR–Cas systems. Nat. Rev. Microbiol..

[35] Li F., Ye Q., Chen M., Xiang X., Zhang J., Pang R., Xue L., Wang J., Gu Q., Lei T. (2021). Cas12aFDet: A CRISPR/Cas12a-based fluorescence platform for sensitive and specific detection of Listeria monocytogenes serotype 4c. Anal. Chim. Acta.

[36] Khan Z., Ali Z., Khan A.A., Sattar T., Zeshan A., Saboor T., Binyamin B. (2022). History and Classification of CRISPR/Cas System.

[37] Javed M.R., Sadaf M., Ahmed T., Jamil A., Nawaz M., Abbas H., Ijaz A. (2018). CRISPR-Cas system: History and prospects as a genome editing tool in microorganisms. Curr. Microbiol..

[38] Huang Z., Fang J., Zhou M., Gong Z., Xiang T. (2022). CRISPR-Cas13: A new technology for the rapid detection of pathogenic microorganisms. Front. Microbiol..

[39] Zhou S., Liu B., Zheng D., Chen L., Yang J. (2025). VFDB 2025: An integrated resource for exploring anti-virulence compounds. Nucleic Acids Res..

[40] Chen L., Yang J., Yu J., Yao Z., Sun L., Shen Y., Jin Q. (2005). VFDB: A reference database for bacterial virulence factors. Nucleic Acids Res..

[41] Soni A., Al-Sarayreh M., Reis M.M., Brightwell G. (2021). Hyperspectral imaging and deep learning for quantification of Clostridium sporogenes spores in food products using 1D-convolutional neural networks and random forest model. Food Res. Int..

[42] Nikzadfar M., Rashvand M., Zhang H., Shenfield A., Genovese F., Altieri G., Matera A., Tornese I., Laveglia S., Paterna G. (2024). Hyperspectral imaging aiding artificial intelligence: A reliable approach for food qualification and safety. Appl. Sci..

[43] Mansuri S.M., Chakraborty S.K., Mahanti N.K., Pandiselvam R. (2022). Effect of germ orientation during Vis-NIR hyperspectral imaging for the detection of fungal contamination in maize kernel using PLS-DA, ANN and 1D-CNN modelling. Food Control.

[44] Kalasinsky K.S., Hadfield T., Shea A.A., Kalasinsky V.F., Nelson M.P., Neiss J., Drauch A.J., Vanni G.S., Treado P.J. (2007). Raman chemical imaging spectroscopy reagentless detection and identification of pathogens: Signature development and evaluation. Anal. Chem..

[45] Jo K., Lee S., Lee D.-H., Jeon H., Jung S. (2024). Hyperspectral imaging–based assessment of fresh meat quality: Progress and applications. Microchem. J..

[46] Ghimpeteanu G., Rajani H., Quintana J., Garcia R. (2025). Hyperspectral Imaging for Identifying Foreign Objects on Pork Belly. arXiv.

[47] Rady A., Adedeji A.A. (2020). Application of hyperspectral imaging and machine learning methods to detect and quantify adulterants in minced meats. Food Anal. Methods.

[48] Ali S., Aslam R., Arshad M.I., Mahmood M.S., Nawaz Z., Abbas R.Z., Khan A. (2021). Meat borne bacterial pathogens. Veterinary Pathobiology and Public Health.

[49] Mackenzie J.S., McKinnon M., Jeggo M. (2014). One Health: From concept to practice. Confronting Emerging Zoonoses: The One Health Paradigm.

[50] Christou L. (2011). The global burden of bacterial and viral zoonotic infections. Clin. Microbiol. Infect..

[51] Espinosa R., Tago D., Treich N. (2020). Infectious diseases and meat production. Environ. Resour. Econ..

[52] Ramees T.P., Dhama K., Karthik K., Rathore R.S., Kumar A., Saminathan M., Tiwari R., Malik Y.S., Singh R.K. (2017). Arcobacter: An emerging food-borne zoonotic pathogen, its public health concerns and advances in diagnosis and control—A comprehensive review. Vet. Q..

[53] Smith J.L., Fratamico P.M. (2018). Emerging and re-emerging foodborne pathogens. Foodborne Pathog. Dis..

[54] Akbar A., Anal A.K. (2011). Food safety concerns and food-borne pathogens, Salmonella, Escherichia coli and Campylobacter. FUUAST J. Biol..

[55] Dodd C. (2017). Infrequent microbial infections. Foodborne Diseases.

[56] Duffy L.L., Fegan N. (2012). Prevalence and concentration of *Arcobacter* spp. on Australian beef carcasses. J. Food Prot..

[57] Cho T.J., Hwang J.Y., Kim H.W., Kim Y.K., Il Kwon J., Kim Y.J., Lee K.W., Kim S.A., Rhee M.S. (2019). Underestimated risks of infantile infectious disease from the caregiver’s typical handling practices of infant formula. Sci. Rep..

[58] Shah A., Saleha A., Murugaiyah M., Zunita Z., Memon A. (2012). Prevalence and distribution of *Arcobacter* spp. in raw milk and retail raw beef. J. Food Prot..

[59] Mardaneh J. (2021). Cronobacter Sakazakii: A Foodborne Pathogenic Bacterium in Immunocompromised and Hospitalized Patients. Intern. Med. Today.

[60] Hartantyo S.H.P., Chau M.L., Koh T.H., Yap M., Yi T., Cao D.Y.H., Gutierrez R.A., Ng L.C. (2020). Foodborne Klebsiella pneumoniae: Virulence potential, antibiotic resistance, and risks to food safety. J. Food Prot..

[61] Deepan G., Bhanu Rekha V., Ajay Kumar V., Kavita Vasudevan P., Quintoil N. (2023). Hygienic practices and prevalence of Klebsiella pneumoniae among butchers in a beef slaughterhouse: A comprehensive study. Pharma Innov. J..

[62] Aslam B., Khurshid M., Arshad M.I., Muzammil S., Rasool M., Yasmeen N., Shah T., Chaudhry T.H., Rasool M.H., Shahid A. (2021). Antibiotic resistance: One health one world outlook. Front. Cell. Infect. Microbiol..

[63] Theocharidi N.A., Balta I., Houhoula D., Tsantes A.G., Lalliotis G.P., Polydera A.C., Stamatis H., Halvatsiotis P. (2022). High prevalence of Klebsiella pneumoniae in Greek meat products: Detection of virulence and antimicrobial resistance genes by molecular techniques. Foods.

[64] Stenfors Arnesen L.P., Fagerlund A., Granum P.E. (2008). From soil to gut: Bacillus cereus and its food poisoning toxins. FEMS Microbiol. Rev..

[65] Gharib A., El-Hamid M., El-Aziz N., Yonan E., Allam M. (2020). Bacillus cereus: Pathogenicity, viability and adaptation. Adv. Anim. Vet. Sci..

[66] Fransen N.G., van den Elzen A.M., Urlings B.A., Bijker P.G. (1996). Pathogenic micro-organisms in slaughterhouse sludge—A survey. Int. J. Food Microbiol..

[67] Gourama H. (2020). Foodborne pathogens. Food Safety Engineering.

[68] Hernández-Cortez C., Palma-Martínez I., Gonzalez-Avila L.U., Guerrero-Mandujano A., Solís R.C., Castro-Escarpulli G. (2017). Food poisoning caused by bacteria (food toxins). Poisoning: From Specific Toxic Agents to Novel Rapid and Simplified Techniques for Analysis.

[69] Zeng H., De Reu K., Gabriël S., Mattheus W., De Zutter L., Rasschaert G. (2021). Salmonella prevalence and persistence in industrialized poultry slaughterhouses. Poult. Sci..

[70] Braz V.S., Melchior K., Moreira C.G. (2020). Escherichia coli as a multifaceted pathogenic and versatile bacterium. Front. Cell. Infect. Microbiol..

[71] Yang S.-C., Lin C.-H., Aljuffali I.A., Fang J.-Y. (2017). Current pathogenic Escherichia coli foodborne outbreak cases and therapy development. Arch. Microbiol..

[72] Jordan K., McAuliffe O. (2018). Listeria monocytogenes in foods. Adv. Food Nutr. Res..

[73] Lund B.M., O’Brien S.J. (2011). The occurrence and prevention of foodborne disease in vulnerable people. Foodborne Pathog. Dis..

[74] Acheson D.W., Lubin L.F. (2008). Vulnerable populations and their susceptibility to foodborne disease. The Microbiological Safety of Food in Healthcare Settings.

[75] Wang Z., Tao X., Liu S., Zhao Y., Yang X. (2021). An update review on Listeria infection in pregnancy. Infect. Drug Resist..

[76] Kumar S.B., Arnipalli S.R., Ziouzenkova O. (2020). Antibiotics in food chain: The consequences for antibiotic resistance. Antibiotics.

[77] Conceição S., Queiroga M.C., Laranjo M. (2023). Antimicrobial resistance in Bacteria from meat and meat products: A one health perspective. Microorganisms.

[78] Patra S.D., Mohakud N.K., Panda R.K., Sahu B.R., Suar M. (2021). Prevalence and multidrug resistance in Salmonella enterica Typhimurium: An overview in South East Asia. World J. Microbiol. Biotechnol..

[79] Frye J.G., Jackson C.R. (2013). Genetic mechanisms of antimicrobial resistance identified in Salmonella enterica, Escherichia coli, and *Enteroccocus* spp. isolated from US food animals. Front. Microbiol..

[80] Costa M.M., Cardo M., Soares P., Cara d’Anjo M., Leite A. (2022). Multi-drug and β-lactam resistance in Escherichia coli and food-borne pathogens from animals and food in Portugal, 2014–2019. Antibiotics.

[81] Algammal A.M., Hetta H.F., Elkelish A., Alkhalifah D.H.H., Hozzein W.N., Batiha G.E.-S., El Nahhas N., Mabrok M.A. (2020). Methicillin-Resistant Staphylococcus aureus (MRSA): One health perspective approach to the bacterium epidemiology, virulence factors, antibiotic-resistance, and zoonotic impact. Infect. Drug Resist..

[82] Pesavento G., Ducci B., Comodo N., Nostro A.L. (2007). Antimicrobial resistance profile of Staphylococcus aureus isolated from raw meat: A research for methicillin resistant Staphylococcus aureus (MRSA). Food Control.

[83] Gupta Y.D., Bhandary S. (2024). Artificial intelligence for understanding mechanisms of antimicrobial resistance and antimicrobial discovery: A new age model for translational research. Artificial Intelligence and Machine Learning in Drug Design and Development.

[84] Nayak D.S.K., Mahapatra S., Routray S.P., Sahoo S., Sahoo S.K., Fouda M.M., Singh N., Isenovic E.R., Saba L., Suri J. (2024). aiGeneR 1.0: An artificial intelligence technique for the revelation of informative and antibiotic resistant genes in *Escherichia coli*. Front. Biosci..

[85] Voogt A.M., Schrijver R.S., Temürhan M., Bongers J.H., Sijm D.T. (2023). Opportunities for regulatory authorities to assess animal-based measures at the slaughterhouse using sensor technology and artificial intelligence: A review. Animals.

[86] Steinkey R., Moat J., Gannon V., Zovoilis A., Laing C. (2020). Application of artificial intelligence to the in silico assessment of antimicrobial resistance and risks to human and animal health presented by priority enteric bacterial pathogens. Can. Commun. Dis. Rep..

[87] Gauba A., Rahman K.M. (2023). Evaluation of antibiotic resistance mechanisms in gram-negative bacteria. Antibiotics.

[88] Kutter E., Kuhl S., Alavidze Z., Blasdel B. (2005). Phage therapy: Bacteriophages as natural, self-limiting antibiotics. Textb. Nat. Med..

[89] Khan M.A.S., Rahman S.R. (2022). Use of phages to treat antimicrobial-resistant Salmonella infections in poultry. Vet. Sci..

[90] Sukumaran A.T., Nannapaneni R., Kiess A., Sharma C.S. (2015). Reduction of Salmonella on chicken meat and chicken skin by combined or sequential application of lytic bacteriophage with chemical antimicrobials. Int. J. Food Microbiol..

[91] Seyfi R., Kahaki F.A., Ebrahimi T., Montazersaheb S., Eyvazi S., Babaeipour V., Tarhriz V. (2020). Antimicrobial peptides (AMPs): Roles, functions and mechanism of action. Int. J. Pept. Res. Ther..

[92] Silveira R.F., Roque-Borda C.A., Vicente E.F. (2021). Antimicrobial peptides as a feed additive alternative to animal production, food safety and public health implications: An overview. Anim. Nutr..

[93] FAO (2020). Gateway to poultry production and products. Food and Agriculture Organisation of the United Nations.

[94] Erdem Büyükkiraz M., Kesmen Z. (2022). Antimicrobial peptides (AMPs): A promising class of antimicrobial compounds. J. Appl. Microbiol..

[95] McGaw L. (2025). Use of plant-derived extracts and bioactive compound mixtures against multidrug resistant bacteria affecting animal health and production. Fighting Multidrug Resistance with Herbal Extracts, Essential Oils and Their Components.

[96] Yuan X., Fan L., Jin H., Wu Q., Ding Y. (2025). Phage engineering using synthetic biology and artificial intelligence to enhance phage applications in food industry. Curr. Opin. Food Sci..

[97] Yan J., Cai J., Zhang B., Wang Y., Wong D.F., Siu S.W.J.A. (2022). Recent progress in the discovery and design of antimicrobial peptides using traditional machine learning and deep learning. Antibiotics.

[98] Petrova D.E. AI-Guided Development of Nanomedicines for Targeting Multidrug-Resistant Bacteria.

[99] Li G., Lin P., Wang K., Gu C.-C., Kusari S. (2022). Artificial intelligence-guided discovery of anticancer lead compounds from plants and associated microorganisms. Trends Cancer.

[100] Váradi L., Luo J.L., Hibbs D.E., Perry J.D., Anderson R.J., Orenga S., Groundwater P.W. (2017). Methods for the detection and identification of pathogenic bacteria: Past, present, and future. Chem. Soc. Rev..

[101] Hilton S., Castro-Nallar E., Pérez-Losada M., Toma I., McCaffrey T., Hoffman E. (2016). Metataxonomic and metagenomic approaches vs. culture-based techniques for clinical pathology. Front. Microbiol..

[102] Chen Y. (2022). Detection of Campylobacter Jejuni Using a Hybrid Paper/Polymer-Based Microfluidic Device Based on the Recombinase Polymerase Amplification and Lateral Flow Assay. Doctoral Dissertation.

[103] Galhoum M., Eed H., Soliman E. (2022). Prevalence, conventional and molecular characterization of Salmonella isolated from chicken farms and slaughterhouses. Adv. Anim. Vet. Sci..

[104] Thapa S. (2024). Detection of Salmonella in Ground Chicken Using SPR Biosensor and Lmmunomagnetic-Chemiluminescent Assay. Master’s Thesis.

[105] Cao L., Zeng L., Wang Y., Cao J., Han Z., Chen Y., Wang Y., Zhong G., Qiao S. (2024). U2-Net and ResNet50-Based Automatic Pipeline for Bacterial Colony Counting. Microorganisms.

[106] Huang L., Wu T. (2018). Novel neural network application for bacterial colony classification. Theor. Biol. Med. Model..

[107] Zheng L., Wen Y., Ren W., Duan H., Lin J., Irudayaraj J. (2022). Hyperspectral dark-field microscopy for pathogen detection based on spectral angle mapping. Sens. Actuators B Chem..

[108] Al-Dulaimi K.A.K., Banks J., Chandran V., Tomeo-Reyes I., Nguyen Thanh K. (2018). Classification of white blood cell types from microscope images: Techniques and challenges. Microscopy Science: Last Approaches on Educational Programs and Applied Research.

[109] Alzahrani A. (2025). A fluorescent Strategy for *Escherichia coli* Detection in Raw Beef in combination with Click Chemistry. J. Food Compos. Anal..

[110] Fuller M.E., Streger S.H., Rothmel R.K., Mailloux B.J., Hall J.A., Onstott T.C., Fredrickson J.K., Balkwill D.L., DeFlaun M.F. (2000). Development of a vital fluorescent staining method for monitoring bacterial transport in subsurface environments. Appl. Environ. Microbiol..

[111] Venkatesh Babu G., Perumal P., Muthu S., Pichai S., Sankar Narayan K., Malairaj S. (2018). Enhanced method for High Spatial Resolution surface imaging and analysis of fungal spores using Scanning Electron Microscopy. Sci. Rep..

[112] Sasaki A. (2022). Recent advances in the standardization of fluorescence microscopy for quantitative image analysis. Biophys. Rev..

[113] Das S., Zun P. (2025). Intelligent Software System for Low-Cost, Brightfield Segmentation: Algorithmic Implementation for Cytometric Auto-Analysis. arXiv.

[114] Cox K.L., Devanarayan V., Kriauciunas A., Manetta J., Montrose C., Sittampalam S. (2019). Immunoassay methods. Assay Guidance Manual [Internet].

[115] Vashist S.K., Luong J.H. (2018). Immunoassays: An overview. Handb. Immunoass. Technol..

[116] Engvall E., Perlmann P. (1971). Enzyme-linked immunosorbent assay (ELISA) quantitative assay of immunoglobulin G. Immunochemistry.

[117] Song C., Li J., Liu J., Liu Q. (2016). Simple sensitive rapid detection of *Escherichia coli* O157: H7 in food samples by label-free immunofluorescence strip sensor. Talanta.

[118] Ahmed S., Ning J., Peng D., Chen T., Ahmad I., Ali A., Lei Z., Abu bakr Shabbir M., Cheng G., Yuan Z. (2020). Current advances in immunoassays for the detection of antibiotics residues: A review. Food Agric. Immunol..

[119] Johnson H., Bukovic J., Kauffmann P. (1973). Staphylococcal enterotoxins A and B: Solid-phase radioimmunoassay in food. Appl. Microbiol..

[120] Lequin R.M. (2005). Enzyme Immunoassay (EIA)/Enzyme-Linked Immunosorbent Assay (ELISA). Clin. Chem..

[121] Chen Y., Wang Z., Liu Y., Wang X., Li Y., Ma P., Gu B., Li H. (2018). Recent advances in rapid pathogen detection method based on biosensors. Eur. J. Clin. Microbiol. Infect. Dis..

[122] Alahi M.E.E., Mukhopadhyay S.C. (2017). Detection methodologies for pathogen and toxins: A review. Sensors.

[123] Polat E.O., Cetin M.M., Tabak A.F., Bilget Güven E., Uysal B.Ö., Arsan T., Kabbani A., Hamed H., Gül S.B. (2022). Transducer technologies for biosensors and their wearable applications. Biosensors.

[124] Knopf G.K., Bassi A.S. (2018). Introduction to Biosensors and Bioelectronics. Smart Biosensor Technology.

[125] Morales M.A., Halpern J.M. (2018). Guide to selecting a biorecognition element for biosensors. Bioconjugate Chem..

[126] Sharma P.S., Iskierko Z., Pietrzyk-Le A., D’Souza F., Kutner W. (2015). Bioinspired intelligent molecularly imprinted polymers for chemosensing: A mini review. Electrochem. Commun..

[127] Karunakaran R., Keskin M. (2022). Biosensors: Components, mechanisms, and applications. Analytical Techniques in Biosciences.

[128] Naresh V., Lee N. (2021). A review on biosensors and recent development of nanostructured materials-enabled biosensors. Sensors.

[129] Massad-Ivanir N., Shtenberg G., Raz N., Gazenbeek C., Budding D., Bos M.P., Segal E. (2016). Porous silicon-based biosensors: Towards real-time optical detection of target bacteria in the food industry. Sci. Rep..

[130] Pohanka M. (2017). The piezoelectric biosensors: Principles and applications, a review. Int. J. Electrochem. Sci..

[131] Chauhan R., Singh J., Solanki P.R., Manaka T., Iwamoto M., Basu T., Malhotra B. (2016). Label-free piezoelectric immunosensor decorated with gold nanoparticles: Kinetic analysis and biosensing application. Sens. Actuators B Chem..

[132] Singh A., Sharma A., Ahmed A., Sundramoorthy A.K., Furukawa H., Arya S., Khosla A. (2021). Recent advances in electrochemical biosensors: Applications, challenges, and future scope. Biosensors.

[133] Helali S., Sawelem Eid Alatawi A., Abdelghani A. (2018). Pathogenic *Escherichia coli* biosensor detection on chicken food samples. J. Food Saf..

[134] Wang L., Lin J. (2020). Recent advances on magnetic nanobead based biosensors: From separation to detection. TrAC Trends Anal. Chem..

[135] Lee S.-Y., Kim U., Kim Y., Lee S.J., Park E.Y., Oh S.-W. (2023). Enhanced detection of Listeria monocytogenes using tetraethylenepentamine-functionalized magnetic nanoparticles and LAMP-CRISPR/Cas12a-based biosensor. Anal. Chim. Acta.

[136] Pauliukaite R., Voitechovič E. (2020). Multisensor Systems and Arrays for Medical Applications Employing Naturally-Occurring Compounds and Materials. Sensors.

[137] Rasheed S., Kanwal T., Ahmad N., Fatima B., Najam-ul-Haq M., Hussain D. (2024). Advances and challenges in portable optical biosensors for onsite detection and point-of-care diagnostics. TrAC Trends Anal. Chem..

[138] Mabhude Y. (2024). Development of Gold Nanoparticles Based Lateral Flow Assay for Detection of Food and Water-Borne Pathogens. Doctoral Dissertation.

[139] Kim Woong-Hee (2021). Development of a Detection System for Food Hazards by Aggregation Between Gold Nanoparticles and Aptamer-Decorated Bifunctional Linkers. Doctoral Dissertation.

[140] Oh S.Y., Heo N.S., Shukla S., Cho H.-J., Vilian A.E., Kim J., Lee S.Y., Han Y.-K., Yoo S.M., Huh Y.S. (2017). Development of gold nanoparticle-aptamer-based LSPR sensing chips for the rapid detection of Salmonella typhimurium in pork meat. Sci. Rep..

[141] Forbes J.D., Knox N.C., Ronholm J., Pagotto F., Reimer A. (2017). Metagenomics: The next culture-independent game changer. Front. Microbiol..

[142] Liu Y., Yu H., Cheng Y., Guo Y., Yao W., Xie Y. (2020). Non-destructive monitoring of Staphylococcus aureus biofilm by surface-enhanced Raman scattering spectroscopy. Food Anal. Methods.

[143] Chindelevitch L., Jauneikaite E., Wheeler N.E., Allel K., Ansiri-Asafoakaa B.Y., Awuah W.A., Bauer D.C., Beisken S., Fan K., Grant G. (2022). Applying data technologies to combat AMR: Current status, challenges, and opportunities on the way forward. arXiv.

[144] Mckillip J.L., Drake M. (2004). Real-time nucleic acid–based detection methods for pathogenic bacteria in food. J. Food Prot..

[145] Perry L., Heard P., Kane M., Kim H., Savikhin S., DomINguez W., Applegate B. (2007). Application of multiplex polymerase chain reaction to the detection of pathogens in food. J. Rapid Methods Autom. Microbiol..

[146] Niessen L., Luo J., Denschlag C., Vogel R.F. (2013). The application of loop-mediated isothermal amplification (LAMP) in food testing for bacterial pathogens and fungal contaminants. Food Microbiol..

[147] Liu M., Hou Y., Cheng Y., Li Z., Zeng J., Li L., Luo J., Shen B.J.I.M. (2025). Recent advances in isothermal amplification techniques coupled with clustered regularly interspaced short palindromic repeat/Cas systems. Interdiscip. Med..

[148] Peruzy M.F., Murru N., Yu Z., Kerkhof P.-J., Neola B., Joossens M., Proroga Y., Houf K. (2019). Assessment of microbial communities on freshly killed wild boar meat by MALDI-TOF MS and 16S rRNA amplicon sequencing. Int. J. Food Microbiol..

[149] Masih N.J.E.P. (2014). Rapid Methods for Detection of Food-Borne Bacterial Pathogens Using Molecular Based Technology. Educ. Plus.

[150] Law J.W.-F., Ab Mutalib N.-S., Chan K.-G., Lee L.-H. (2015). Rapid methods for the detection of foodborne bacterial pathogens: Principles, applications, advantages and limitations. Front. Microbiol..

[151] Ishino Y., Krupovic M., Forterre P. (2018). History of CRISPR-Cas from encounter with a mysterious repeated sequence to genome editing technology. J. Bacteriol..

[152] Wang S., Fan Y., Feng Z., Song M., Li Q., Jiang B., Qin F., Liu H., Lan L., Yang M. (2021). Rapid nucleic acid detection of *Escherichia coli* O157: H7 based on CRISPR/Cas12a system. Food Control.

[153] Miller R.R., Montoya V., Gardy J.L., Patrick D.M., Tang P. (2013). Metagenomics for pathogen detection in public health. Genome Med..

[154] Haverkamp T.H., Spilsberg B., Johannessen G.S., Torp M., Sekse C. (2024). Detection and characterization of Campylobacter in air samples from poultry houses using shot-gun metagenomics–a pilot study. BMC Microbiol..

[155] Puiu M., Bala C. (2020). Microfluidics-integrated biosensing platforms as emergency tools for on-site field detection of foodborne pathogens. TrAC Trends Anal. Chem..

[156] Bai Z., Xu X., Wang C., Wang T., Sun C., Liu S., Li D. (2022). A comprehensive review of detection methods for *Escherichia coli* O157: H7. TrAC Trends Anal. Chem..

[157] Cinquanta L., Fontana D.E., Bizzaro N. (2017). Chemiluminescent immunoassay technology: What does it change in autoantibody detection?. Autoimmun. Highlights.

[158] Deng Z., Yun Y.-H., Duan N., Wu S. (2025). Artificial intelligence algorithms-assisted biosensors in the detection of foodborne pathogenic bacteria: Recent advances and future trends. Trends Food Sci. Technol..

[159] Aliev T.A., Lavrentev F.V., Dyakonov A.V., Diveev D.A., Shilovskikh V.V., Skorb E.V. (2024). Electrochemical platform for detecting *Escherichia coli* bacteria using machine learning methods. Biosens. Bioelectron..

[160] Ma L., Yi J., Wisuthiphaet N., Earles M., Nitin N. (2023). Accelerating the detection of bacteria in food using artificial intelligence and optical imaging. Appl. Environ. Microbiol..

[161] Öz Y.Y., Sönmez Ö.İ., Karaman S., Öz E., Unal C.B., Karataş A.Y. (2020). Rapid and sensitive detection of Salmonella spp. in raw minced meat samples using droplet digital PCR. Eur. Food Res. Technol..

[162] Singh J., Birbian N., Sinha S., Goswami A. (2014). A critical review on PCR, its types and applications. Int. J. Adv. Res. Biol. Sci..

[163] Baker M., Zhang X., Maciel-Guerra A., Dong Y., Wang W., Hu Y., Renney D., Hu Y., Liu L., Li H. (2023). Machine learning and metagenomics reveal shared antimicrobial resistance profiles across multiple chicken farms and abattoirs in China. Nat. Food.

[164] Cooper A.L., Wong A., Tamber S., Blais B.W., Carrillo C.D. (2024). Analysis of Antimicrobial Resistance in Bacterial Pathogens Recovered from Food and Human Sources: Insights from 639,087 Bacterial Whole-Genome Sequences in the NCBI Pathogen Detection Database. Microorganisms.

[165] Brown E., Dessai U., McGarry S., Gerner-Smidt P. (2019). Use of whole-genome sequencing for food safety and public health in the United States. Foodborne Pathog. Dis..

[166] Trachsel J.M., Bearson B.L., Brunelle B.W., Bearson S.M. (2022). Relationship and distribution of Salmonella enterica serovar I 4,[5], 12: I:-strain sequences in the NCBI Pathogen Detection database. BMC Genom..

[167] Cernava T., Rybakova D., Buscot F., Clavel T., McHardy A.C., Meyer F., Meyer F., Overmann J., Stecher B., Sessitsch A. (2022). Metadata harmonization-Standards are the key for a better usage of omics data for integrative microbiome analysis. Environ. Microbiome.

[168] Gensheimer K., Allard M.W., Timme R.E., Brown E., Hintz L., Pettengill J., Strain E., Tallent S.M., Vélez L.F., King E. (2025). Genomic Surveillance of Foodborne Pathogens: Advances and Obstacles. J. Public Health Manag. Pract..

[169] Yan S., Liu X., Li C., Jiang Z., Li D., Zhu L. (2022). Genomic virulence genes profile analysis of *Salmonella enterica* isolates from animal and human in China from 2004 to 2019. Microb. Pathog..

[170] AlJindan R., AlEraky D.M., Farhat M., Almandil N.B., AbdulAzeez S., Borgio J.F. (2023). Genomic Insights into Virulence Factors and Multi-Drug Resistance in Clostridium perfringens IRMC2505A. Toxins.

[171] de Lagarde M., Vanier G., Arsenault J., Fairbrother J.M.J.A. (2021). High risk clone: A proposal of criteria adapted to the one health context with application to enterotoxigenic *Escherichia coli* in the pig population. Antibiotics.

[172] Sun J., Yin H., Ju C., Wang Y., Yang Z.J.G. (2024). DTVF: A User-Friendly Tool for Virulence Factor Prediction Based on ProtT5 and Deep Transfer Learning Models. Genes.

[173] Dyer N.P., Päuker B., Baxter L., Gupta A., Bunk B., Overmann J., Diricks M., Dreyer V., Niemann S., Holt K.E. (2025). EnteroBase in 2025: Exploring the genomic epidemiology of bacterial pathogens. Nucleic Acids Res..

[174] Achtman M., Zhou Z., Charlesworth J., Baxter L. (2022). EnteroBase: Hierarchical clustering of 100 000s of bacterial genomes into species/subspecies and populations. Philos. Trans. R. Soc. B Biol. Sci..

[175] Zhou Z., Charlesworth J., Achtman M. (2021). HierCC: A multi-level clustering scheme for population assignments based on core genome MLST. Bioinformatics.

[176] Zhang L., Guo W., Lv C. (2024). Modern technologies and solutions to enhance surveillance and response systems for emerging zoonotic diseases. Sci. One Health.

[177] Cookson A.L., Marshall J.C., Biggs P.J., Rogers L.E., Collis R.M., Devane M., Stott R., Wilkinson D.A., Kamke J., Brightwell G.J.A. (2022). Whole-genome sequencing and virulome analysis of *Escherichia coli* isolated from New Zealand environments of contrasting observed land use. Appl. Environ. Microbiol..

[178] Benedict K.M., Reses H., Vigar M., Roth D.M., Roberts V.A., Mattioli M., Cooley L.A., Hilborn E.D., Wade T.J., Fullerton K.E. (2017). Surveillance for Waterborne Disease Outbreaks Associated with Drinking Water-United States, 2013-2014. MMWR Morb. Mortal. Wkly. Rep..

[179] Centers for Disease Control and Prevention (2016). Vital Signs: Disparities in Tobacco-Related Cancer Incidence and Mortality—United States, 2004–2013. MMWR Morb. Mortal. Wkly. Rep..

[180] Canning M., Birhane M.G., Dewey-Mattia D., Lawinger H., Cote A., Gieraltowski L., Schwensohn C., Tagg K.A., Watkins L.K.F., Robyn M.P. (2023). Salmonella outbreaks linked to beef, United States, 2012–2019. J. Food Prot..

[181] Timme R.E., Sanchez Leon M., Allard M.W. (2019). Utilizing the public GenomeTrakr database for foodborne pathogen traceback. Foodborne Bacterial Pathogens: Methods and Protocols.

[182] Cebeci T., Tanrıverdi E.S., Otlu B. (2024). A first study of meat-borne enterococci from butcher shops: Prevalence, virulence characteristics, antibiotic resistance and clonal relationship. Vet. Res. Commun..

[183] Gilbert J.M., White D.G., McDermott P.F. (2007). The US national antimicrobial resistance monitoring system. Future Microbiol..

[184] Jones T.F., Scallan E., Angulo F.J. (2007). FoodNet: Overview of a decade of achievement. Foodborne Pathog. Dis..

[185] Baranyi J., Tamplin M.L. (2004). ComBase: A common database on microbial responses to food environments. J. Food Prot..

[186] Gonzalez M.G. (2021). Evaluating the Effect of Inoculation Method and Validating Existing Combase Models for Listeria Monocytogenes on Ten Whole Intact Raw Fruits and Vegetables. Master’s Thesis.

[187] Walker L., Sun S., Thippareddi H. (2023). Growth comparison and model validation for growth of Shiga toxin-producing *Escherichia coli* (STEC) in ground beef. LWT.

[188] Sayers E.W., Beck J., Bolton E.E., Bourexis D., Brister J.R., Canese K., Comeau D.C., Funk K., Kim S., Klimke W. (2021). Database resources of the national center for biotechnology information. Nucleic Acids Res..

[189] Sayers E.W., Cavanaugh M., Clark K., Pruitt K.D., Sherry S.T., Yankie L., Karsch-Mizrachi I. (2023). GenBank 2023 update. Nucleic Acids Res..

[190] Nelson K.E., Fouts D.E., Mongodin E.F., Ravel J., DeBoy R.T., Kolonay J.F., Rasko D.A., Angiuoli S.V., Gill S.R., Paulsen I.T. (2004). Whole genome comparisons of serotype 4b and 1/2a strains of the food-borne pathogen Listeria monocytogenes reveal new insights into the core genome components of this species. Nucleic Acids Res..

[191] Karp P.D., Billington R., Caspi R., Fulcher C.A., Latendresse M., Kothari A., Keseler I.M., Krummenacker M., Midford P.E., Ong Q. (2019). The BioCyc collection of microbial genomes and metabolic pathways. Brief. Bioinform..

[192] Nwadiugwu M.C., Monteiro N. (2023). Applied genomics for identification of virulent biothreats and for disease outbreak surveillance. Postgrad. Med. J..

[193] Peng J., Xiao R., Wu C., Zheng Z., Deng Y., Chen K., Xiang Y., Xu C., Zou L., Liao M. (2024). Characterization of the prevalence of Salmonella in different retail chicken supply modes using genome-wide and machine-learning analyses. Food Res. Int..

[194] Friede A., Reid J.A., Ory H.W. (1993). CDC WONDER: A comprehensive on-line public health information system of the Centers for Disease Control and Prevention. Am. J. Public Health.

[195] Timme R.E., Rand H., Sanchez Leon M., Hoffmann M., Strain E., Allard M., Roberson D., Baugher J.D. (2018). GenomeTrakr proficiency testing for foodborne pathogen surveillance: An exercise from 2015. Microb. Genom..

[196] Benson D.A., Cavanaugh M., Clark K., Karsch-Mizrachi I., Lipman D.J., Ostell J., Sayers E.W. (2012). GenBank. Nucleic Acids Res..

[197] Karanth S. (2021). Development of Machine Learning and Advanced Data Analytical Techniques to Incorporate Genomic Data in Predictive Modeling for Salmonella Enterica. Doctoral Dissertation.

[198] Alsulimani A., Akhter N., Jameela F., Ashgar R.I., Jawed A., Hassani M.A., Dar S.A. (2024). The impact of artificial intelligence on microbial diagnosis. Microorganisms.

[199] Stocker M.D., Pachepsky Y.A., Hill R.L. (2022). Prediction of *Escherichia coli* concentrations in agricultural pond waters: Application and comparison of machine learning algorithms. Front. Artif. Intell..

[200] Oniciuc E.A., Likotrafiti E., Alvarez-Molina A., Prieto M., Santos J.A., Alvarez-Ordóñez A. (2018). The present and future of whole genome sequencing (WGS) and whole metagenome sequencing (WMS) for surveillance of antimicrobial resistant microorganisms and antimicrobial resistance genes across the food chain. Genes.

[201] Rantsiou K., Kathariou S., Winkler A., Skandamis P., Saint-Cyr M.J., Rouzeau-Szynalski K., Amézquita A. (2018). Next generation microbiological risk assessment: Opportunities of whole genome sequencing (WGS) for foodborne pathogen surveillance, source tracking and risk assessment. Int. J. Food Microbiol..

[202] den Besten H., Duqué B., Williams M. (2019). Challenges in Campylobacter detection and control. IAFP 2019 Annual Meeting Abstracts.

[203] Belias A., Bolten S., Wiedmann M. (2024). Challenges and opportunities for risk-and systems-based control of Listeria monocytogenes transmission through food. Compr. Rev. Food Sci. Food Saf..

[204] Mu W., Kleter G.A., Bouzembrak Y., Dupouy E., Frewer L.J., Radwan Al Natour F.N., Marvin H. (2024). Making food systems more resilient to food safety risks by including artificial intelligence, big data, and internet of things into food safety early warning and emerging risk identification tools. Compr. Rev. Food Sci. Food Saf..

[205] Zulli A., Zhang Z., Ruedaflores M., Sahly J., Angel D., Rohatgi K., Malik W., Hao R., Shepherd J., Peccia J. (2025). Utilizing Internet Search Trends and Wastewater Surveillance to Identify Infectious Disease Outbreaks in Communities. Environ. Sci. Technol..

[206] Nastasijević I., Moračanin S.V. (2021). Digitalization in the meat chain. Acta Agric. Serbica.

[207] Mehta A., Rathod T., Swaminarayan P.R. (2021). Application of COVID-19 Pandemic Using Artificial Intelligence. Artificial Intelligence for COVID-19.

[208] Yi J., Wisuthiphaet N., Raja P., Nitin N., Earles J.M. (2023). AI-enabled biosensing for rapid pathogen detection: From liquid food to agricultural water. Water Res..

[209] Li Y., Huang R., Zhu J., Teng Y., Liu B., Lyu Z., Chen T., Li Y., Li Y., Huang R. (2023). Bacterial infection. Radiology of Infectious and Inflammatory Diseases.

[210] Onyeaka H., Akinsemolu A., Miri T., Nnaji N.D., Emeka C., Tamasiga P., Pang G., Al-sharify Z. (2024). Advancing food security: The role of machine learning in pathogen detection. Appl. Food Res..

[211] Ma H., Li G., Zhang H., Wang X., Li F., Yan J., Hong L., Zhang Y., Pu Q. (2025). Rapid and ultra-sensitive detection of foodborne pathogens by deep learning-enhanced microfluidic biosensing. Sens. Actuators B Chem..

[212] Jia Z., Luo Y., Wang D., Holliday E., Sharma A., Green M.M., Roche M.R., Thompson-Witrick K., Flock G., Pearlstein A.J. (2024). Surveillance of pathogenic bacteria on a food matrix using machine-learning-enabled paper chromogenic arrays. Biosens. Bioelectron..

[213] Yan S., Liu C., Fang S., Ma J., Qiu J., Xu D., Li L., Yu J., Li D., Liu Q. (2020). SERS-based lateral flow assay combined with machine learning for highly sensitive quantitative analysis of *Escherichia coli* O157: H7. Anal. Bioanal. Chem..

[214] Zhang B., Rahman M.A., Liu J., Huang J., Yang Q. (2023). Real-time detection and analysis of foodborne pathogens via machine learning based fiber-optic Raman sensor. Measurement.

[215] Ciloglu F.U., Caliskan A., Saridag A.M., Kilic I.H., Tokmakci M., Kahraman M., Aydin O. (2021). Drug-resistant Staphylococcus aureus bacteria detection by combining surface-enhanced Raman spectroscopy (SERS) and deep learning techniques. Sci. Rep..

[216] Qi Y., Hu D., Jiang Y., Wu Z., Zheng M., Chen E.X., Liang Y., Sadi M.A., Zhang K., Chen Y.P. (2023). Recent progresses in machine learning assisted Raman spectroscopy. Adv. Opt. Mater..

[217] Ciloglu F.U., Saridag A.M., Kilic I.H., Tokmakci M., Kahraman M., Aydin O. (2020). Identification of methicillin-resistant Staphylococcus aureus bacteria using surface-enhanced Raman spectroscopy and machine learning techniques. Analyst.

[218] Garcia-Vozmediano A., Maurella C., Ceballos L.A., Crescio E., Meo R., Martelli W., Pitti M., Lombardi D., Meloni D., Pasqualini C. (2024). Machine learning approach as an early warning system to prevent foodborne Salmonella outbreaks in northwestern Italy. Vet. Res..

[219] Wang H., Cui W., Guo Y., Du Y., Zhou Y. (2021). Machine learning prediction of foodborne disease pathogens: Algorithm development and validation study. JMIR Med. Inform..

[220] Sarkar P.R. (2025). Artificial Intelligence Based Models for Predicting Foodborne Pathogen Risk In Public Health Systems. Int. J. Bus. Econ. Insights.

[221] Kim S., Lee M.H., Wiwasuku T., Day A.S., Youngme S., Hwang D.S., Yoon J.-Y. (2021). Human sensor-inspired supervised machine learning of smartphone-based paper microfluidic analysis for bacterial species classification. Biosens. Bioelectron..

[222] Zhang J., Li C., Rahaman M.M., Yao Y., Ma P., Zhang J., Zhao X., Jiang T., Grzegorzek M. (2022). A comprehensive review of image analysis methods for microorganism counting: From classical image processing to deep learning approaches. Artif. Intell. Rev..

[223] Qazi R.A., Aman N., Ullah N., Jamila N., Bibi N. (2024). Recent advancement for enhanced *E. coli* detection in electrochemical biosensors. Microchem. J..

[224] Wang L., Huo X., Jiang F., Xi X., Li Y., Lin J. (2023). Dual-functional manganese dioxide nanoclusters for power-free microfluidic biosensing of foodborne bacteria. Sens. Actuators B Chem..

[225] Man Y., Ban M., Jin X., Li A., Tao J., Pan L. (2023). An integrated distance-based microfluidic aptasensor for visual quantitative detection of Salmonella with sample-in-answer-out capability. Sens. Actuators B Chem..

[226] Khan F.M., Gupta R., Sekhri S. (2021). A convolutional neural network approach for detection of *E. coli* bacteria in water. Environ. Sci. Pollut. Res..

[227] Wu X., Yuan Z., Gao S., Zhang X., El-Mesery H.S., Lu W., Dai X., Xu R. (2025). Electrochemical Biosensors Driving Model Transformation for Food Testing. Foods.

[228] Prempeh N.Y.A., Nunekpeku X., Kutsanedzie F.Y., Murugesan A., Li H. (2025). A Comprehensive Review of Non-Destructive Monitoring of Food Freshness and Safety Using NIR Spectroscopy and Biosensors: Challenges and Opportunities. Chemosensors.

[229] Mazur F., Han Z., Tjandra A.D., Chandrawati R. (2024). Digitalization of colorimetric sensor technologies for food safety. Adv. Mater..

[230] Yang M., Liu X., Luo Y., Pearlstein A.J., Wang S., Dillow H., Reed K., Jia Z., Sharma A., Zhou B. (2021). Machine learning-enabled non-destructive paper chromogenic array detection of multiplexed viable pathogens on food. Nat. Food.

[231] Jia Z., Lin Z., Luo Y., Cardoso Z.A., Wang D., Flock G.H., Thompson-Witrick K.A., Yu H., Zhang B. (2024). Enhancing pathogen identification in cheese with high background microflora using an artificial neural network-enabled paper chromogenic array sensor approach. Sens. Actuators B Chem..

[232] Feng L., Wu B., Zhu S., He Y., Zhang C. (2021). Application of visible/infrared spectroscopy and hyperspectral imaging with machine learning techniques for identifying food varieties and geographical origins. Front. Nutr..

[233] Sun H. (2022). Image target detection and recognition method using deep learning. Adv. Multimed..

[234] Yan S., Wang S., Qiu J., Li M., Li D., Xu D., Li D., Liu Q. (2021). Raman spectroscopy combined with machine learning for rapid detection of food-borne pathogens at the single-cell level. Talanta.

[235] Bai Z., Du D., Zhu R., Xing F., Yang C., Yan J., Zhang Y., Kang L. (2024). Establishment and comparison of in situ detection models for foodborne pathogen contamination on mutton based on SWIR-HSI. Front. Nutr..

[236] Hassan S.A., Khalil M.A., Auletta F., Filosa M., Camboni D., Menciassi A., Oddo C.M. (2023). Contamination detection using a deep convolutional neural network with safe machine—Environment interaction. Electronics.

[237] Medus L.D., Saban M., Francés-Víllora J.V., Bataller-Mompeán M., Rosado-Muñoz A. (2021). Hyperspectral image classification using CNN: Application to industrial food packaging. Food Control.

[238] Wu K., Ji Z., Wang H., Shao X., Li H., Zhang W., Kong W., Xia J., Bao X. (2025). A Comprehensive Review of AI Methods in Agri-Food Engineering: Applications, Challenges, and Future Directions. Electronics.

[239] Ledesma D., Symes S., Richards S. (2021). Advancements within modern machine learning methodology: Impacts and prospects in biomarker discovery. Curr. Med. Chem..

[240] Satria B., Afrianto N., Ningsih L., Sakinah P., Sidauruk A., Mayola L. (2025). Comparative Analysis of Weighted-KNN, Random Forest, and Support Vector Machine Models for Beef and Pork Image Classification Using Machine Learning. JOIV Int. J. Inform. Vis..

[241] Yıldız B.İ., Karabağ K. (2025). Prediction of Beef Production Using Linear Regression, Random Forest and k-Nearest Neighbors Algorithms. Tarim Doga Derg..

[242] Saberioon M., Císař P., Labbé L., Souček P., Pelissier P., Kerneis T. (2018). Comparative performance analysis of support vector machine, random forest, logistic regression and k-nearest neighbours in rainbow trout (oncorhynchus mykiss) classification using image-based features. Sensors.

[243] Mustapha A., Ishak I., Zaki N.N.M., Ismail-Fitry M.R., Arshad S., Sazili A.Q. (2024). Application of machine learning approach on halal meat authentication principle, challenges, and prospects: A review. Heliyon.

[244] Yan C. (2025). A review on spectral data preprocessing techniques for machine learning and quantitative analysis. iScience.

[245] Çetin V., Yıldız O. (2022). A comprehensive review on data preprocessing techniques in data analysis. Pamukkale Üniv. Mühendis. Bilim. Derg..

[246] Ràfols P., Vilalta D., Brezmes J., Cañellas N., Del Castillo E., Yanes O., Ramírez N., Correig X. (2018). Signal preprocessing, multivariate analysis and software tools for MA (LDI)-TOF mass spectrometry imaging for biological applications. Mass Spectrom. Rev..

[247] Hsu L.L., Culhane A.C. (2020). Impact of data preprocessing on integrative matrix factorization of single cell data. Front. Oncol..

[248] Teodorescu V., Obreja Brașoveanu L. (2025). Assessing the validity of k-fold cross-validation for model selection: Evidence from bankruptcy prediction using random forest and XGBoost. Computation.

[249] Steyerberg E.W., Bleeker S.E., Moll H.A., Grobbee D.E., Moons K.G. (2003). Internal and external validation of predictive models: A simulation study of bias and precision in small samples. J. Clin. Epidemiol..

[250] Chen Z., Zhang G., Zhang F. (2026). Multimodal AI for Real-Time Food Safety and Quality: From Sensors to Foundation Models, Edge Deployment, and Regulation. Food Sci. Nutr..

[251] Sowmya T., Anita E.M. (2023). A comprehensive review of AI based intrusion detection system. Meas. Sens..

[252] Fernandez E.I., Ferreira A.S., Cecílio M.H.M., Chéles D.S., de Souza R.C.M., Nogueira M.F.G., Rocha J.C. (2020). Artificial intelligence in the IVF laboratory: Overview through the application of different types of algorithms for the classification of reproductive data. J. Assist. Reprod. Genet..

[253] Razavi-Termeh S.V., Sadeghi-Niaraki A., Jelokhani-Niaraki M., Choi S.-M. (2024). Exploring multi-pollution variability in the urban environment: Geospatial AI-driven modeling of air and noise. Int. J. Digit. Earth.

[254] Xie Z., He F., Fu S., Sato I., Tao D., Sugiyama M. (2021). Artificial neural variability for deep learning: On overfitting, noise memorization, and catastrophic forgetting. Neural Comput..

[255] Naser M., Alavi A.H. (2023). Error metrics and performance fitness indicators for artificial intelligence and machine learning in engineering and sciences. Archit. Struct. Constr..

[256] Agrawal K., Goktas P., Kumar N., Leung M.-F. (2025). Artificial intelligence in personalized nutrition and food manufacturing: A comprehensive review of methods, applications, and future directions. Front. Nutr..

[257] Rahman M.H.-U., Sikder R., Tripathi M., Zahan M., Ye T., Gnimpieba Z.E., Jasthi B.K., Dalton A.B., Gadhamshetty V. (2024). Machine learning-assisted raman spectroscopy and SERS for bacterial pathogen detection: Clinical, food safety, and environmental applications. Chemosensors.

[258] Olufemi O.I., Ayeni O., Olagoke-Komolafe O.E. (2024). Advancing real-time predictive systems for listeria and *Escherichia coli* detection in meat processing facilities across the USA. Int. J. Multidiscip. Res. Growth Eval..

[259] Lewis N.L. (2012). Analysis of Simulated Outbreak Data and Spatial Analysis of Highly Pathogenic Avian Influenza for Preparedness Planning and Policy. Master’s Thesis.

[260] Jiang Q., Mo Q., Ge C., Li W., Mai J., Chen Y., Liu Y., Deng X., Yang Z., Wang D. (2025). Applications of artificial intelligence-driven microfluidics in medical laboratory science. Interdiscip. Med..

[261] Wang H., Ceylan Koydemir H., Qiu Y., Bai B., Zhang Y., Jin Y., Tok S., Yilmaz E.C., Gumustekin E., Rivenson Y. (2020). Early detection and classification of live bacteria using time-lapse coherent imaging and deep learning. Light Sci. Appl..

[262] Qian S., Cui Y., Cai Z., Li L. (2022). Applications of smartphone-based colorimetric biosensors. Biosens. Bioelectron. X.

[263] Cho I.-H., Ku S. (2017). Current technical approaches for the early detection of foodborne pathogens: Challenges and opportunities. Int. J. Mol. Sci..

[264] Nastasijevic I., Kundacina I., Jaric S., Pavlovic Z., Radovic M., Radonic V. (2025). Recent advances in biosensor technologies for meat production chain. Foods.

[265] Milios K.T., Drosinos E.H., Zoiopoulos P.E. (2014). Food Safety Management System validation and verification in meat industry: Carcass sampling methods for microbiological hygiene criteria–A review. Food Control.

[266] Risalvato J., Sewid A.H., Eda S., Gerhold R.W., Wu J.J. (2025). Strategic Detection of *Escherichia coli* in the Poultry Industry: Food Safety Challenges, One Health Approaches, and Advances in Biosensor Technologies. Biosensors.

[267] Ghovvati S., Nassiri M., Mirhoseini S., Moussavi A.H., Javadmanesh A. (2009). Fraud identification in industrial meat products by multiplex PCR assay. Food Control.

[268] Ilhak O.I., Arslan A. (2007). Identification of meat species by polymerase chain reaction (PCR) technique. Turk. J. Vet. Anim. Sci..

[269] Kumar Y., Bansal S., Jaiswal P. (2017). Loop-mediated isothermal amplification (LAMP): A rapid and sensitive tool for quality assessment of meat products. Compr. Rev. Food Sci. Food Saf..

[270] Singh P.K., Jairath G., Ahlawat S.S., Pathera A., Singh P. (2016). Biosensor: An emerging safety tool for meat industry. J. Food Sci. Technol..

[271] Biglia A., Barge P., Tortia C., Comba L., Aimonino D.R., Gay P. (2022). Artificial intelligence to boost traceability systems for fraud prevention in the meat industry. J. Agric. Eng..

[272] Gorbunova N.A., Nikitina M.A. (2026). The potential of artificial intelligence in the meat industry. Theory Pract. Meat Process..

[273] Unnevehr L.J., Jensen H.H. (1996). HACCP as a regulatory innovation to improve food safety in the meat industry. Am. J. Agric. Econ..

[274] Jutzi S. (2004). Good Practices for the Meat Industry.

